# Notch signaling determines cell-fate specification of the two main types of vomeronasal neurons of rodents

**DOI:** 10.1242/dev.200448

**Published:** 2022-07-04

**Authors:** Raghu Ram Katreddi, Ed Zandro M. Taroc, Sawyer M. Hicks, Jennifer M. Lin, Shuting Liu, Mengqing Xiang, Paolo E. Forni

**Affiliations:** 1 Department of Biological Sciences, University at Albany, State University of New York, Albany, NY 12222, USA; 2 The RNA Institute, University at Albany, State University of New York, Albany, NY 12222, USA; 3 The Center for Neuroscience Research, University at Albany, State University of New York, Albany, NY 12222, USA; 4 State Key Laboratory of Ophthalmology, Zhongshan Ophthalmic Center, Sun Yat-sen University, Guangzhou 510060, China

**Keywords:** Single cell RNA sequencing, Vomeronasal organ, Notch signaling, Dll4, Neuronal dichotomy, Mouse, Neuronal differentiation

## Abstract

The ability of terrestrial vertebrates to find food and mating partners, and to avoid predators, relies on the detection of chemosensory information. Semiochemicals responsible for social and sexual behaviors are detected by chemosensory neurons of the vomeronasal organ (VNO), which transmits information to the accessory olfactory bulb. The vomeronasal sensory epithelium of most mammalian species contains a uniform vomeronasal system; however, rodents and marsupials have developed a more complex binary vomeronasal system, containing vomeronasal sensory neurons (VSNs) expressing receptors of either the V1R or V2R family. In rodents, V1R/apical and V2R/basal VSNs originate from a common pool of progenitors. Using single cell RNA-sequencing, we identified differential expression of Notch1 receptor and Dll4 ligand between the neuronal precursors at the VSN differentiation dichotomy. Our experiments show that Notch signaling is required for effective differentiation of V2R/basal VSNs. In fact, Notch1 loss of function in neuronal progenitors diverts them to the V1R/apical fate, whereas Notch1 gain of function redirects precursors to V2R/basal. Our results indicate that Notch signaling plays a pivotal role in triggering the binary differentiation dichotomy in the VNO of rodents.

## Abstract (precis)

**Summary:** Single cell RNA sequencing in the vomeronasal organ reveals a crucial role for Dll4-Notch1 signaling in the formation of V1R^+^/apical and V2R^+^/basal vomeronasal sensory neurons of rodents.

## INTRODUCTION

Neural stem/progenitor cells can give rise to multiple neuronal cell types that differ in gene expression, function and neuronal connectivity. Investigating the molecular mechanisms that establish different neuronal cell fates is crucial to understand how neuronal systems evolve and form and to identify mechanisms underlying neurodevelopmental disorders (Livesey and Cepko, 2001; Louvi and Artavanis-Tsakonas, 2006; Bonnefont and Vanderhaeghen, 2021). The vomeronasal organ (VNO) is a specialized chemosensory organ that, in many vertebrate species, is located at the base of the nasal cavity (Levy et al., 2020). The VNO is responsible for the detection of semiochemicals, molecules that can trigger stereotypical mating/sex behaviors, parental behaviors and predator avoidance (Stowers et al., 2002; He et al., 2008; Papes et al., 2010; Flanagan et al., 2011; Trouillet et al., 2021). Most of the vertebrates with a functional VNO have a uniform vomeronasal (VN) system with vomeronasal sensory neurons (VSNs) expressing receptors of the V1R family (Grus et al., 2005). However, rodents and some marsupials have developed a more complex binary VN system including a second type of VSNs expressing receptors of the V2R family (Takigami et al., 2004). In mice, the V1R neurons (often referred to as apical VSNs) express the Gαi2 G protein subunit (encoded by *Gnai2*), the Meis2 transcription factor and are mostly distributed in the apical territories of vomeronasal sensory epithelium (VSE) (Enomoto et al., 2011). Conversely, the V2R neurons (called basal VSNs) express the Gαo G protein subunit (encoded by *Gnao1*), the Tfap2e (AP-2ε) transcription factor and are, for the most part, located in basal regions of the VSE and around the vasculature (Grus and Zhang, 2004; Young and Trask, 2007; Enomoto et al., 2011; Lin et al., 2018; Katreddi and Forni, 2021). Apical and basal VSNs detect distinct types of ligands, connect to different areas of the accessory olfactory bulb (AOB) and control distinct behaviors (Chamero et al., 2011; Oboti et al., 2014; Mohrhardt et al., 2018; Trouillet et al., 2019; Pallé et al., 2020).

In mice, VSN neurogenesis starts during embryonic development at around embryonic day (E) 11.5 and continues throughout life, starting from a limited number of progenitors in the marginal zones of the VNO (Cau et al., 2002; Martinez-Marcos et al., 2005; De La Rosa-Prieto et al., 2009; Taroc et al., 2020; Katreddi and Forni, 2021). How the cell fate of apical and basal VSN types is established is not fully understood. A pivotal study by Enomoto and coworkers previously showed that the transcription factor Bcl11b plays a key role in controlling the correct establishment of apical and basal VSNs in the developing VNO (Enomoto et al., 2011). However, which extrinsic signaling pathways control the expression of Bcl11b in the VNO has not been investigated. Moreover, the same study identified the transcription factor Tfap2e as a potential target of Bcl11b and suggested a role for Tfap2e in controlling basal neuron differentiation. In a follow-up study, we proposed that Tfap2e is not responsible for initiating the apical versus basal identity bifurcation but is instead crucial for the expression of several genes defining, and maintaining, basal VSN molecular identity (Lin et al., 2018). In fact, we observed that after Tfap2e loss of function, basal VSNs lose their expression of basal genes (*Gnao1* and V2Rs) and switch to expressing apical genes such as *Gnai2* and V1Rs (Lin et al., 2018).

In this study, we adopted a single cell sequencing strategy to investigate the mechanisms involved in directing cell fate specification and differentiation of apical and basal VSNs. By following the transcriptomic profile of postnatal stem cells/progenitors and immature VSNs, we identified differential expression of Notch1 receptor and Dll4 ligand across Neurog1^+^/Neurod1^+^ VSN precursors. We then adopted both loss-of-function and gain-of-function *in vivo* strategies to test the role of the Notch signaling pathway in establishing VSN binary differentiation fates. Genetic experiments confirmed differential effects of loss and gain of Notch signaling on the switch between apical and basal VSN cell fate, with Notch loss of function directing cells towards apical fate and gain of function towards basal fate. Our data characterizing the signaling responsible for more complex VNO development offer a new context in which Notch-mediated cell fate choice is a conserved strategy for establishing binary cellular diversification and increasing the neuronal repertoire in developing neuroepithelia.

## RESULTS

### Single cell profiling of the whole adult VNO identifies VSN dichotomy

**Fig. 1.**
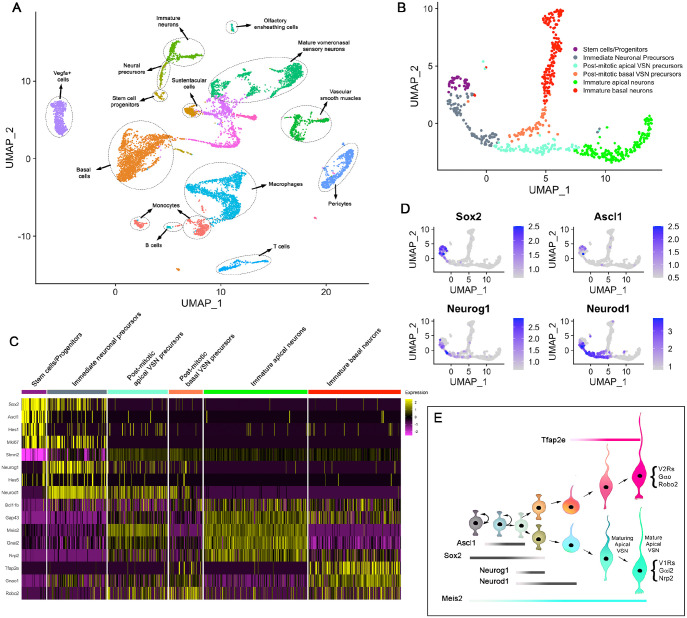
**scRNA-seq identified adult neurogenesis and VSN apical-basal dichotomy in the mouse VNO.** (A) UMAP dimensional reduction plot of Seurat object 1 shows neuronal and non-neuronal cell clusters of the VNO. Each colored cluster of cells corresponds to an identified cell type that has a similar transcriptomic profile. (B) Seurat object 2 generated from stem cell progenitors, neural precursors and immature neurons of Seurat object 1 identifies the VSN apical-basal dichotomy. (C) Heatmap of known gene expression that is specific to each stage of VSN formation. (D) Feature plots of *Sox2*, *Ascl1*, *Neurog1* and *Neurod1* in Seurat object 2. (E) Summary schematic depicting dynamic expression of stage-specific transcription factors throughout apical and basal VSN differentiation.

**Fig. 2.**
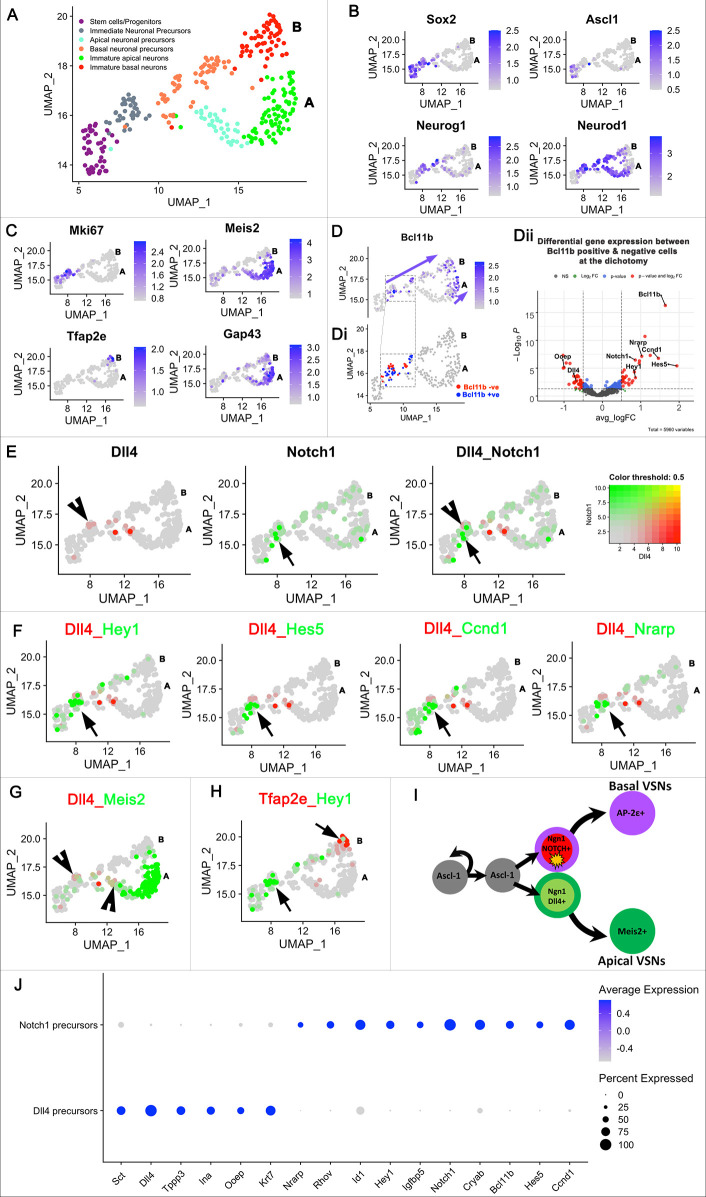
**scRNA-seq analysis identified Notch1-Dll4 signaling in the VSN dichotomy.** (A) UMAP dimension plot of Seurat object3 specifically focusing on the VSN apical-basal split. Letters A and B on the right of the UMAP denote apical and basal VSN branches, respectively. (B,C) Feature plots of known marker genes specific to neuronal progenitors, precursors and VSNs. (D) Feature plot of *Bcl11b* expression across the VSN dichotomy. The arrows highlight continuous *Bcl11b* expression in the basal VSN branch. *Bcl11b* expression appears at a later stage in the apical VSN branch. (Di) Feature plot highlighting *Bcl11b*^+^ and *Bcl11b*^−^ cells at the split point used for differential gene expression analysis. (Dii) Volcano plot highlighting *Notch*-related genes differentially expressed between *Bcl11b*^+^ and *Bcl11b*^−^ clusters. (E) Feature plots of *Dll4* (arrowhead) and *Notch1* (arrow) demonstrate their mutually exclusive expression at the VSN dichotomy. (F) Co-expression feature plots of *Dll4* and downstream Notch signaling targets like *Hes5*, *Hey1*, *Nrarp* and *Ccnd1* (arrows) show that active Notch signaling occurs transiently at the split point and early stages of the basal VSN trajectory. (G) Co-expression feature plot of *Dll4* versus *Meis2* highlights *Dll4* expression at the start of the apical VSN branch. (H) Co-expression feature plot of *Hey1* versus *Tfap2e* shows active Notch signaling in the basal VSN branch. (I) Schematic showing the inferred Notch1-Dll4 signaling required for establishing the early VSN dichotomy. (J) Dot plot of a few selected genes that are differentially expressed between the *Dll4*^+^ and *Notch1*^+^ clusters at the VSN dichotomy.

In mice, adult neurogenesis occurs throughout life at the marginal zones of the VSE (Giacobini et al., 2000; Martinez-Marcos et al., 2005). To identify multiple cell types of the VNO including stem cells, VSNs and non-neuronal cell types, we performed single cell RNA-sequencing (scRNA-seq) from whole VNO. We dissociated single cells from a total of five male mice at postnatal day (P) 60 for the sequencing. For the analysis, we filtered out low-quality cells and performed clustering and analysis of the scRNA-seq data using Seurat (Stuart et al., 2019). A total of 10,582 single cell transcriptomes passed quality control measures. Based on the expression of the top 2000 highly variable features across the population, cells were clustered in Seurat object 1 and visualized using uniform manifold approximation projection (UMAP) (McInnes, et al., 2018) (Fig. 1A). We identified neuronal and non-neuronal cell types in the VNO and annotated clusters based on the following gene expression: basal cells (*Trp63*, *Krt5*), stem cell progenitors (*Sox2*, *Ascl1*), neural precursors (*Neurog1*, *Neurod1*), immature neurons (*Gap43*), mature VSNs (*Omp*), sustentacular cells (*Fezf2*, *Cyp2a5*), olfactory ensheathing cells (*S100b*, *Plp1*, *Mpz*, *Sox10*), pericytes (*Pecam1*, *Eng*, *Sox17*), vascular smooth muscle cells (*Tagln*, *Acta2*), Vegfa^+^ cells (*Vegfa*), T cells (*Cd3d*, *Cd3e*), B cells (*Cd19*, *Cd79a*), macrophages (*C1qa*, *C1qb*), monocytes (*Chil3*, *Clec10a*, *Ccr2*) (Fig. 1A; Fig. S1). We focused our further clustering and analysis on stem cell progenitors, neural precursors and immature neurons with the goal of characterizing the mechanisms underlying VSN cell fate specification. We subset these clusters into Seurat object 2 (Fig. 1B) and re-clustered based on highly variable features among the selected cells. The expression pattern of known apical- and basal-VSN specific genes (Fig. 1C,E) further corroborated the scRNA-seq subclustering (Enomoto et al., 2011; Lin et al., 2018; Taroc et al., 2020). Interestingly, *Sox2* expression was found to extend from the Ascl1^+^ progenitor stage cells to Neurog1^+^ and some Neurod1^+^ neuronal precursor-stage cells (Figs 1D and 2B), similar to the findings in the main olfactory epithelium (Guo et al., 2010; Packard et al., 2011). In addition, we found that *Neurod1* expression, which coincides with *Neurog1* in the initial expression, is retained beyond the dichotomy splitting apical and basal VSN lineages (Figs 1D and 2B). We labeled Ki67^+^/Neurog1^+^/Neurod1^+^ cells before the dichotomy as immediate neuronal precursors, whereas Ki67^−^/Neurod1^+^ cells in apical and basal lineages are considered as post-mitotic apical and basal VSN precursors, respectively (Fig. 1B,C).

A pseudotime reconstruction was generated by implementing Monocle single cell trajectory analysis on Seurat object 2 (Trapnell et al., 2014; Qiu et al., 2017). In this analysis, we chose Ascl1^+^ neuronal progenitors as a root node (less differentiated stage), and the rest of the single cells in Seurat object 2 were ordered in an unbiased way along the pseudotime. This revealed the formation of a differentiation branched trajectory. This analysis confirmed the validity of the VSN dichotomy obtained from initial Seurat analysis (Fig. S1).

### Differential gene expression identifies the expression of Notch signaling-related genes at the VSN dichotomy

To further focus on the origin of the VSN dichotomy, we re-clustered Ascl1^+^ progenitors and VSN neuronal precursors to form Seurat object 3 (Fig. 2A). Feature plots of Ascl1, Sox2, Neurog1 and Neurod1 support the temporal transcriptional cascade as neuronal progenitors proliferate and differentiate into immediate neuronal precursors (Fig. 2B). Further analysis showed, as expected (Enomoto et al., 2011; Lin et al., 2018), specific expression of the transcription factor *Meis2* in post-mitotic (Ki67^−^) cells acquiring apical VSN identity and specific *Tfap2e* expression in maturing (Gap43^+^) basal VSNs (Fig. 2C). This confirms previous results (Lin et al., 2018), as the expression of the basal-specific gene *Tfap2e* appears at later stages of maturation compared with apical-specific *Meis2*. Given a previous study (Enomoto et al., 2011) suggesting that *Bcl11b* is a key gene controlling cell fate choice of apical and basal VSNs, we assessed the spatial expression pattern of *Bcl11b* in this cell population (Fig. 2D). Initial *Bcl11b* expression was seen at Neurog1/Neurod1 differentiation stages post Ascl1 expression. On the basal VSN trajectory, *Bcl11b* expression occurred as a continuum from the dichotomy throughout the immature VSN stage whereas, on the apical VSN trajectory, *Bcl11b* expression was not detected until early stages of maturation (Fig. 2D, arrows). Based on this observation, we manually clustered Bcl11b^+^ and Bcl11b^−^ cells specifically at the point of separation (dashed boxed area in Fig. 2D and Di). Performing differential gene expression analysis between Bcl11b^+^ and Bcl11b^−^ cells specifically at this point (Fig. 2Di), we found Notch1 receptor to be enriched in the Bcl11b^+^ cluster, whereas Dll4, which is a Notch ligand, was enriched in the Bcl11b^−^ cluster (Fig. 2Dii).

Moreover, we also identified *Hes5*, *Hey1*, *Nrarp* and *Ccnd1* as genes enriched in the Bcl11b^+^ cluster (Fig. 2Dii), genes which, along with *Bcl11b*, are known to be downstream Notch signaling targets (Cau et al., 2000; Ronchini and Capobianco, 2001; Fischer et al., 2004; Li et al., 2010; Jarrett et al., 2019). Spatial colocalization plots of *Notch1* and *Dll4* reinforced their mutually exclusive cellular expression even before the apical-basal dichotomy was established (Fig. 2E). Even though *Notch1* mRNA could be detected in both apical and basal maturing VSNs after the split (Fig. 2E), Notch downstream targets including *Hey1*, *Hes5*, *Nrarp* and *Ccnd1* appeared to be expressed only in Notch1^+^ but not Dll4^+^ cells before the VSN bifurcation and in the early stage basal VSN trajectory (Fig. 2F). In addition, *Dll4* ligand expression co-occurred along with *Meis2*, the apical-specific VSN marker, whereas downstream Notch signaling target *Hey1* was instead expressed along the basal VSN trajectory before *Tfap2e* (Fig. 2G,H). Notably, the expression of other known Notch receptors and ligands within the neuronal precursors was found to be minimal (Fig. S2). We also found that, many cells that are negative for Notch downstream target genes at the split point are positive for expression of the negative regulator of Notch signaling, *Numb*, suggestive of post-translational Notch degradation after the split (Fig. S3) (McGill and McGlade, 2003; McGill et al., 2009). These gene expression patterns suggest that Notch1 signaling is selectively activated in the cells acquiring basal VSN identity during the establishment of the apical versus basal dichotomy, but not after (Fig. 2I,J)

**Fig. 3.**
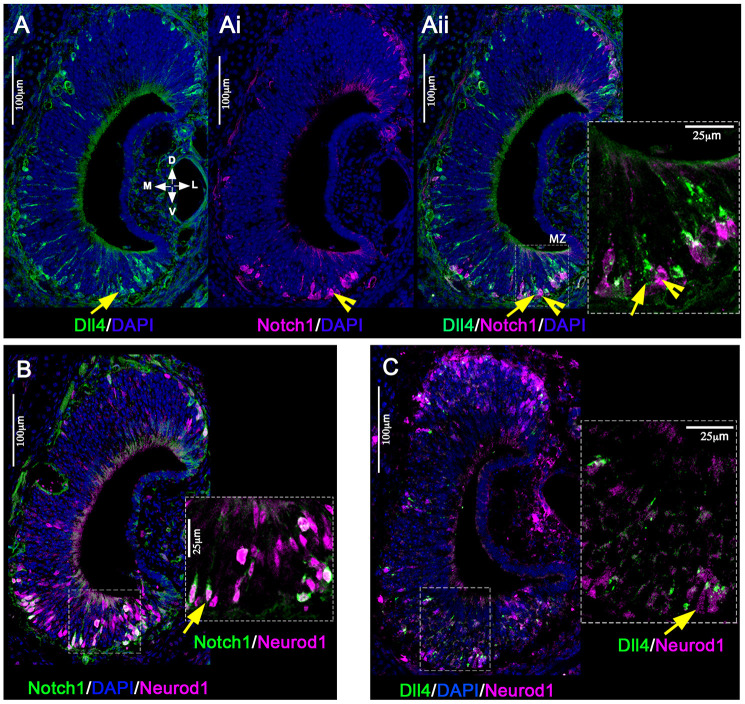
**Notch1 and Dll4 immunoreactivity in Neurod1^+^ cells.** (A-Aii) Immunofluorescence anti Dll4 (green) and Notch1 (magenta) shows expression of Notch ligand and receptor in marginal zones (MZ) and basal regions of the VNO at P1. Inset shows Dll4^+^ (arrow) and Notch1^+^ (arrowhead) cells in close proximity. (B) Immunofluorescence anti Notch1 (green) and Neurod1 (magenta). Notch1 expression co-occurs in the Neurod1^+^ stage (see arrow in the magnification). (C) Immunofluorescence anti Dll4 (green) and Neurod1 (magenta). Dll4 ligand expression occurs in the Neurod1^+^ stage (see arrow in the magnification). D, dorsal; L, lateral; M, medial; V, ventral.

To further validate the occurrence of differential Notch-Delta expression in developing mouse VSNs, we performed immunofluorescence staining for Dll4, Notch1 and Neurod1 on vomeronasal tissue at P1. Imaging confirmed the presence of immunodetectable Notch1 and Delta4 limited to Neurod1^+^ precursors in the VNO (Fig. 3A-C). This analysis also highlighted the proximity of Dll4^+^ and Notch1^+^ cells. These data suggest that differential Delta-Notch expression and activation of Notch signaling in newly formed VSN precursors could play a role in establishing a binary outcome in the differentiation of VSNs. Although we obtained scRNA-seq data from adult mice, we chose to further validate and probe Notch1-Dll4 signaling at early postnatal age to take advantage of the higher rate of neurogenesis (Brann and Firestein, 2010). Notably, scRNA-seq analysis at P10 showed conserved developmental trajectories of apical and basal VSNs as we described at P60 (Fig. S4). An in depth bioinformatic analysis of P10 VNO scRNA-seq data has also been reported elsewhere (Lin et al., 2022 preprint).

### Conditional lineage tracing confirms the formation of Notch1^+^ and Dll4^+^ cells from Ascl1^+^ progenitors

**Fig. 4.**
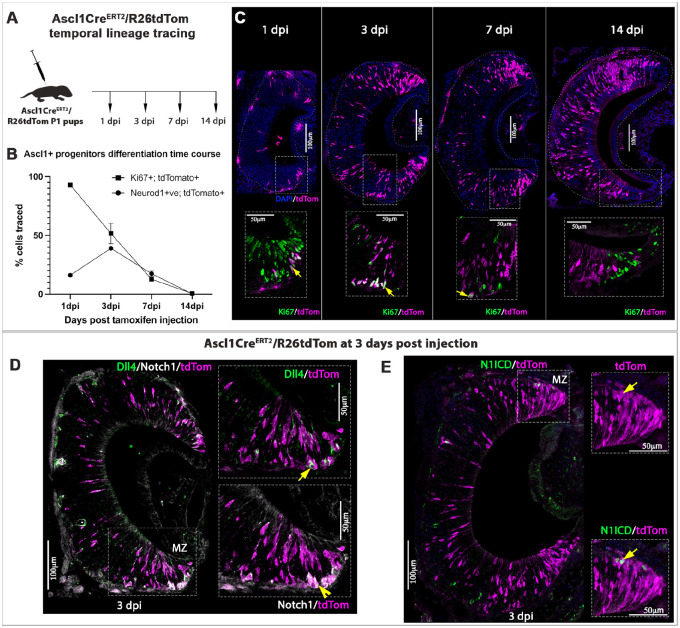
**Ascl1 lineage tracing reveals that both Dll4^+^ and Notch1^+^ cells are progeny of Ascl1^+^ cells.** (A) Ascl1Cre^ERT2^/R26tdTom pups were injected with tamoxifen at P1 and perfused at 1, 3, 7 and 14 days post injection (dpi). (B) Plot showing differentiation time course of Ascl1^+^ traced neuronal progenitors. Data points are percentage tdTom^+^ traced cells that are Ki67^+^ and Neurod1^+^, quantified at different stages. (C) Top panel reflects the increase in tdTom^+^ traced cells from 1 dpi to 14 dpi. Bottom panel shows immunofluorescence anti Ki67 and tdTom. Arrows indicate proliferative traced cells. (D) Immunofluorescence anti Dll4, Notch1 and tdTom in Ascl1Cre^ERT2^ lineage-traced pups at 3 dpi. Inset shows both Dll4^+^ (arrow) and Notch1^+^ (arrowhead) cells in the marginal zone (MZ) that colocalized with tdTom tracing. (E) Immunofluorescence anti Notch intracellular domain (NICD) and tdTom in Ascl1Cre^ERT2^ lineage-traced pups at 3 dpi. Inset shows tdTom^+^ cells colocalized with NICD staining in the MZ (arrow). *n*=3 biological replicates. Data shown as mean±s.e.m.

Temporally controlled genetic lineage tracing of stem cells/progenitors can be used to follow proliferation and differentiation dynamics (Hsu, 2015). We performed conditional lineage tracing of Ascl1Cre^ERT2^/R26tdTom P1 pups and collected VNOs at 1, 3, 7 and 14 days post injection (dpi) (Fig. 4A). We performed immunostaining against Ki67, Neurod1 and tdTom to determine how long it takes for Ascl1 progenitors to differentiate and become post mitotic, and whether Ascl1^+^ progenitors give rise to both Dll4^+^ and Notch1^+^ VSN precursors.

After a single tamoxifen injection, we observed that the number of Ascl1Cre^ERT2^ traced cells increased steadily from 30.3±2.5 cells at 1 dpi to 142.1±14.3 cells at 3 dpi, 225.1±33.7 cells at 7 dpi and 199.8±10.8 cells at 14 dpi (mean±s.e.m.). Notably, proliferation decreased with age, as ∼88% of the traced cells at 7 dpi and a near totality at 14 dpi were post-mitotic (Fig. 4B). These data suggest that Ascl1^+^ transit amplifying cells (Kim et al., 2007) of the VNO undergo proliferation before becoming post-mitotic neurons (Fig. 4C).

Analyzing the expression of Neurod1, which is a marker of cells undergoing differentiation (Figs 1D and 2B), we observed that Neurod1^+^Ascl1^−^ traced cells transiently increased from 16±1.5% at 1 dpi to 39±0.3% at 3 dpi before decreasing to 17±2.2% at 7 dpi and 0.3±0.1% at 14 dpi (mean±s.e.m.) as the cells start to mature (Fig. 4B). These results together indicate that by 1-2 weeks after the initiation of Ascl1 lineage tracing, most of the cells have committed to neuronal differentiation.

We further performed Dll4/Notch1/tdTom immunofluorescence staining after Ascl1Cre^ERT2^/R26tdTom tracing at 3 dpi (Fig. 4D). As expected, we saw Dll4^+^/tdTom^+^ and Notch1^+^/tdTom^+^ double positive cells mostly in the marginal and basal zones of the VSE, where most of the postnatal neurogenesis takes place. Moreover, staining for cleaved Notch intracellular domain (NICD) confirmed the presence of Ascl1^+^ traced progeny undergoing active Notch signaling at 3 dpi (Fig. 4E). In summary, these results suggest that Ascl1^+^ progenitors divide to give rise to Neurod1^+^ precursors that transiently express either Dll4 ligand or Notch1 receptor and are competent for active Notch signaling.

### Loss of active Notch signaling leads maturing VSNs to default to the apical cell fate

**Fig. 5.**
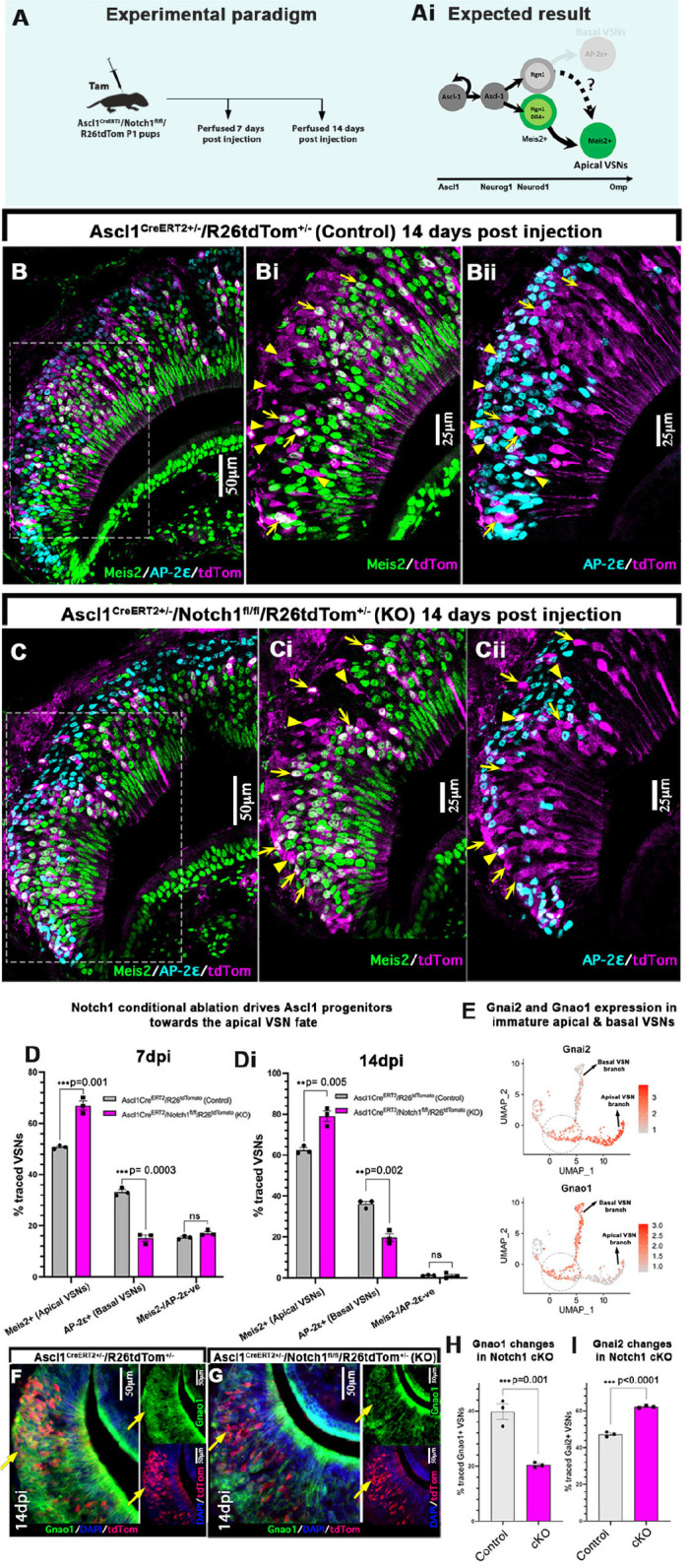
**Notch1 loss of function biases VSN differentiation to the apical fate.** (A) Ascl1Cre^ERT2^/Notch1^fl/fl^/R26tdTom and Ascl1Cre^ERT2^/R26tdTom pups were injected with tamoxifen at P1 and perfused at 7 dpi. (Ai) Expected results with Notch1 receptor KO driving the progenitors towards the apical VSN fate. (B-Cii) Immunofluorescence anti Meis2, Tfap2e (AP-2ε) and tdTom in Ascl1Cre^ERT2^ induced control and Notch1 cKO pups at 14 dpi. Arrows highlight traced Meis2^+^ and arrowheads highlight Tfap2e^+^ VSNs. (D,Di) Quantification of the percentage of traced VSNs that are Meis2^+^, Tfap2e^+^, and Meis2/Tfap2e double negative cells in control and Notch1 KO mice at 7 dpi and 14 dpi. (E) Feature plots of *Gnai2* (*Gαi2*) and *Gnao1* (*Gαo*) genes in the Seurat object 2 show that both G proteins are transcriptionally active in both VSN branches at the early stages of the dichotomy before getting restricted to apical versus basal VSN branches, respectively (see dotted circle). (F,G) Immunofluorescence of *Gnao1*/tdTom in control and *Notch1* cKOs at 14 dpi. Arrows indicate Gnao1 expression in traced cells. (H,I) Quantification of the percentage of traced VSNs that are *Gnao1*^+^ and *Gnai2*^+^ in control and *Notch1* KO mice at 14 dpi. D, Di, H, and I, statistical analysis based on arcsine-transformed values of the percentage data; unpaired two-tailed *t*-test; *n*=3 biological replicates. Data shown as mean±s.e.m. At 7 dpi, the average number of tdTom^+^ cells in control group was 234.2±9.2 and in cKOs it was 189.4±8. At 14 dpi, the average number of tdTom^+^ cells in control group was 229.6±11 and in cKOs it was 195.6±22.7.

To directly test the role of Notch signaling in controlling the differentiation of VSNs, we conditionally knocked out Notch1 receptor from the Ascl1^+^ progenitor stage onwards. We used the Ascl1Cre^ERT2^ driver, as both *Notch1* and *Dll4* expression start immediately after the Ascl1^+^ stage (see Fig. 2B,E). We induced Cre recombination at P1 in both Ascl1Cre^ERT2^/R26tdTom^+/−^ controls and Ascl1Cre^ERT2^/Notch1^fl/fl^/R26tdTom^+/−^ KO pups and analyzed cell phenotypes at 7 dpi (Fig. 5A). In control pups, 1 week after tamoxifen injection, 50.7±0.4% of the traced cells were positive for the apical VSN marker Meis2, 33.2±0.8% cells were positive for basal VSN marker Tfap2e and 15.4±0.4% of traced cells did not yet express either Meis2 or Tfap2e (mean±s.e.m.) (Fig. 5D). In the Ascl1Cre^ERT2^/Notch1 conditional KOs (cKO), 1 week after recombination the Meis2^+^ apical population significantly increased to 66.9±1.7%, whereas the Tfap2e^+^ basal population decreased to 15.17±1.1% and the rest of the 17.1±0.6% traced cells did not express either markers (mean±s.e.m.) (Fig. 5D). In order to follow the cell fate decision of the Meis2/Tfap2e double negative population, we also analyzed the Ascl1Cre^ERT2^/Notch1 KO pups at 14 dpi (Fig. 5B,C). Two weeks after recombination, we still detected a higher number of Meis2^+^/apical cells in the cKOs; however, the population of Meis2/Tfap2e double negative cells was reduced to ∼1% of the total traced cells. These data suggest that most of the double negative cells found at 7 dpi matured to either apical or basal neurons (Fig. 5Di).

In line with previous observations (Enomoto et al., 2011; Lin et al., 2018), our scRNA-seq data show that newly formed apical and basal neurons first co-express *Gnao1* and *Gnai2* and then, over time, establish either Gαi2/apical or Gαo/basal identity (Fig. 5E). By staining sections of Ascl1Cre^ERT2^/Notch1 KO and Ascl1Cre^ERT2^/R26tdTom^+/−^ controls at 14 dpi against Gαo we observed a significantly smaller percentage of Gαo traced cells in cKOs (Fig. 5F-H). Similarly, after immunostaining against Gαi2 we observed an increase in the percentage of cells with apical features. (Fig. 5I). These results overall suggest that active Notch signaling, via the Notch1 receptor, is necessary for the activation of the basal differentiation program.

We then analyzed the effects of induction of conditional Notch1 ablation at a later developmental stage by using Neurog1Cre^ERT2^/Notch1^fl/fl^/R26tdTom^+/−^. We did this to understand whether Notch1 loss of function at Neurog1 stage could still deviate the cell fate towards the apical VSNs. We injected tamoxifen at P1 in both Neurog1Cre^ERT2^/R26tdTom^+/−^ and Neurog1Cre^ERT2^/Notch1^fl/fl^/R26tdTom^+/−^ pups and perfused after 1 week to determine the VSN cell fate switch (Fig. S5). At 7 dpi, Meis2/Tfap2e/tdTom immunofluorescence analysis showed no significant changes in the proportion of Meis2^+^ versus Tfap2e^+^ VSNs in the cKOs when compared with controls (Fig. S5B-D). These data suggest that activation of Notch1 signaling has the ability to define VSN fate in a restricted developmental window, as the Notch1 cKO at later stages has no effect on the cell fate.

The role of Notch signaling in cell fate specification is conserved in many neuronal and non-neuronal systems (Duarte et al., 2004; Kiernan et al., 2005; Del Barrio et al., 2007; Koch et al., 2008). Studies in retina and spinal cord determined that the forkhead transcription factor Foxn4 has a role in inducing Notch1/Dll4-mediated signaling (Del Barrio et al., 2007; Luo et al., 2012; Misra et al., 2014). In both systems, it has been shown that Foxn4 can control the expression of Dll4 and that Foxn4 mutants have aberrant cell fate phenotypes (Del Barrio et al., 2007; Luo et al., 2012). In line with this, co-expression of *Foxn4* and Dll4 in scRNA-seq analysis and immunofluorescence staining at E14 VNO suggested a potential role for Foxn4 in VSN differentiation (Fig. S6). However, we found out that *Foxn4* null mutants maintained detectable Dll4 expression at E14 and we saw no obvious changes in the apical-basal VSN ratio. These data suggest that, in the VNO, Foxn4 is dispensable for the induction of *Dll4* expression.

### The basal VSN differentiation program is established via canonical Notch1 signaling

**Fig. 6.**
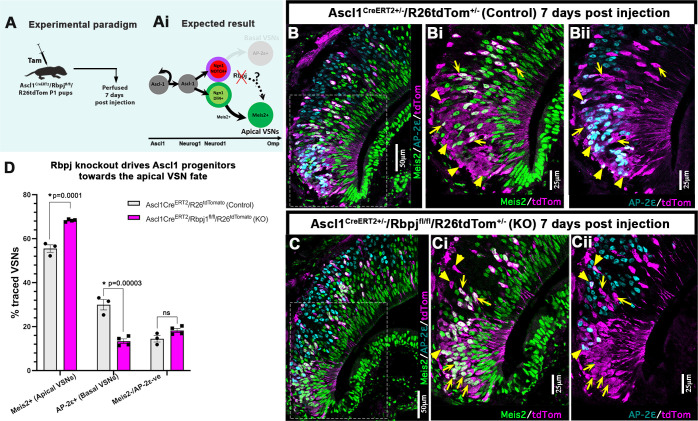
**Canonical Notch signaling establishes basal VSN fate.** (A) Ascl1Cre^ERT2^/Rbpj^fl/fl^/R26tdTom and Ascl1Cre^ERT2^/R26tdTom pups were injected with tamoxifen at P1 and perfused at 7 dpi. (Ai) Expected results with *Rbpj* KO driving the progenitors towards the apical VSN fate. (B-Cii) Immunofluorescence anti Meis2, Tfap2e and tdTom in Ascl1Cre^ERT2^-induced control pups and Rbpj KO pups at 7 dpi. Arrows highlight traced Meis2^+^ and arrowheads highlight traced Tfap2e^+^ basal VSNs. (D) Quantification of the percentage of traced VSNs that are Meis2^+^, Tfap2e^+^, and Meis2/Tfap2e double negative cells in control and *Rbpj* KO mice at 7 dpi. Statistical analysis based on arcsine-transformed values of the percentage data; unpaired two-tailed *t*-test; *n*=3,4 biological replicates. Data shown as mean±s.e.m. At 7 dpi, the average number of tdTom traced cells in control group was 217.5±25.7 and in cKO it was 177±13.9.

Notch signaling can happen either via canonical or non-canonical pathways (Ayaz and Osborne, 2014; Siebel and Lendahl, 2017). In the canonical pathway, once NICD is cleaved after interaction with the ligand^+^ signal sending cell, it translocates to the nucleus to form a transcriptional activation complex with Rbpj, MAML1 and other co-activator factors (Ayaz and Osborne, 2014; Contreras-Cornejo et al., 2016). To test whether basal VSN cell fate determination is occurring via canonical Notch pathway, we conditionally knocked out Rbpj protein using Ascl1Cre^ERT2^ driver at P1 and collected pups 1 week after recombination (Fig. 6A). At 7 dpi, Meis2/Tfap2e/tdTom immunofluorescence analysis revealed an ∼13% increase in Meis2^+^/apical VSNs in the Ascl1Cre^ERT2^/Rbpj^fl/fl^/R26tdTom^+/−^ cKOs compared with Ascl1Cre^ERT2^/R26tdTom^+/−^ control pups. In line with this, Tfap2e^+^/basal population was smaller in the conditional *Rbpj* KOs. This is similar to what we observed after Notch1 ablation. We also found a proportion of Meis2/Tfap2e double negative cells in both control and *Rbpj* KOs at 7 dpi (Fig. 6B-D). The highly overlapping results obtained after Notch1 and Rbpj ablation suggest a prominent role of canonical Notch signaling in establishing the basal VSN program.

### Ascl1Cre^ERT2^-induced Notch1 deletion redirects the cell fate of precursors without changing proliferation or cell death rate

**Fig. 7.**
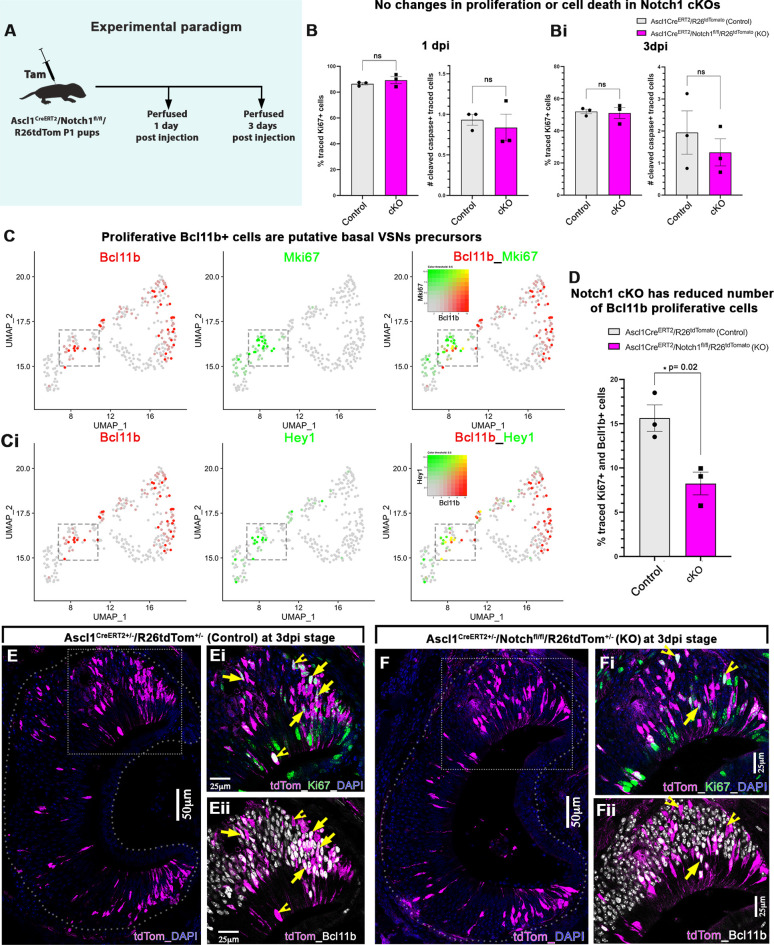
***Notch1* conditional knockout at Ascl1 stage did not change proliferation or cell death at early stages.** (A) Ascl1Cre^ERT2^/Notch1^fl/fl^/R26tdTom and Ascl1Cre^ERT2^/R26tdTom pups were injected with tamoxifen at P1 and perfused at 1 dpi and 3 dpi. (B,Bi) Quantification of percentage traced tdTom^+^ cells undergoing proliferation and quantification of the number of traced tdTom^+^ cells undergoing apoptosis in control and *Notch1* cKOs at 1 dpi and 3 dpi. (C,Ci) Blended feature plots of *Bcl11b*/*Mki67* and *Bcl11b*/*Hey1* show that *Bcl11b*^+^ cells expressed early in the VSN dichotomy (highlighted in the box) are still proliferative and positive for downstream Notch signaling targets like *Hey1*, suggesting they are putative basal VSN precursors. (D) Quantification of percentage traced tdTom^+^ cells that are Bcl11b and Ki67 double positive at 3 dpi. (E-Fii) Immunofluorescence anti tdTom/Ki67/Bcl11b in control and conditional Notch1 KO at 3 dpi. Arrows highlight traced Ki67/Bcl11b double positive cells, whereas notched arrowheads highlight traced Ki67^+^/Bcl11b^−^ cells. In B, Bi and D, Ki67^+^ traced cells in distinct genetic backgrounds were compared as a percentage. Statistical analysis based on arcsine-transformed values of the percentage data; unpaired two-tailed *t*-test; *n*=3 biological replicates. Data shown as mean±s.e.m. At 1 dpi, the average number of tdTom^+^ cells in the control group was 27±2.5 and in cKO it was 33±4.8. At 3 dpi, the average number of tdTom^+^ cells in the control group was 119.5±2.9 and in cKO it was 95.8±11.9.

Notch signaling has a well-established role in proliferation and differentiation of neuronal progenitors (Artavanis-Tsakonas et al., 1999). To test whether the increase in percentage of apical VSN in the Ascl1Cre^ERT2^-driven *Notch1* cKOs is sculpted by changes in proliferation or cell death, we analyzed Ascl1Cre^ERT2^/R26tdTom^+/−^ controls and Ascl1Cre^ERT2^/Notch1^fl/fl^/R26tdTom^+/−^ KO pups at 1 day and 3 days post tamoxifen injection (Fig. 7A). Neither Ki67/tdTom nor cleaved caspase3 (cc3)/tdTom immunofluorescence staining showed any significant changes in rate of proliferation or cell death among traced cells (Fig. 7B).

However, we decided to investigate whether we could detect any early changes in the differentiation trajectory of the proliferative precursors in *Notch1* cKOs. For this, we analyzed Bcl11b expression, as this is an early marker for basal differentiation and a target of Notch transcription activation complex (Li et al., 2010; Enomoto et al., 2011) (Fig. 2B-D, Fig. 7C). Interestingly, at 3 dpi we found a significant reduction in proliferative Bcl11b cells (Ki67/Bcl11b/tdTom triple-positive cells) in *Notch1* cKO pups compared with controls (Fig. 7D-F). These data suggest that Ascl1Cre^ERT2^-driven Notch1 ablation does not affect the overall proliferation or cell death, but prevents Bcl11b expression, which, as previously observed in *Bcl11b* KO (Enomoto et al., 2011), redirects the cell fates towards the Meis2^+^/apical fate.

### Conditional induction of NICD at the Ascl1 progenitor stage leads to sustentacular cell formation

Our results upon ablation of Notch1 suggest that, without Notch signaling, VSN progenitors default towards the apical VSN cell fate. To further test whether active Notch signaling is sufficient to initiate the basal VSN genetic program, we conditionally overexpressed NICD at the Ascl1^+^ stage, using Ascl1Cre^ERT2^/R26NICD^+/−^ pups. We aimed to test whether activation of Notch signaling would redirect all the recombined NICD^+^ cells towards the basal VSN phenotype. In the inducible experiment, we counted GFP^+^ cells to follow NICD recombined cells, as these mice have Cre^ERT2^-activated NICD along with nuclear-localized EGFP reporter (Murtaugh et al., 2003). There were fewer total EGFP^+^ NICD recombined cells compared with control Ascl1Cre^ERT2^/R26tdTom tracing, which may be because of low efficiency in R26NICD recombination, as previously reported (Cheung et al., 2018).

**Fig. 8.**
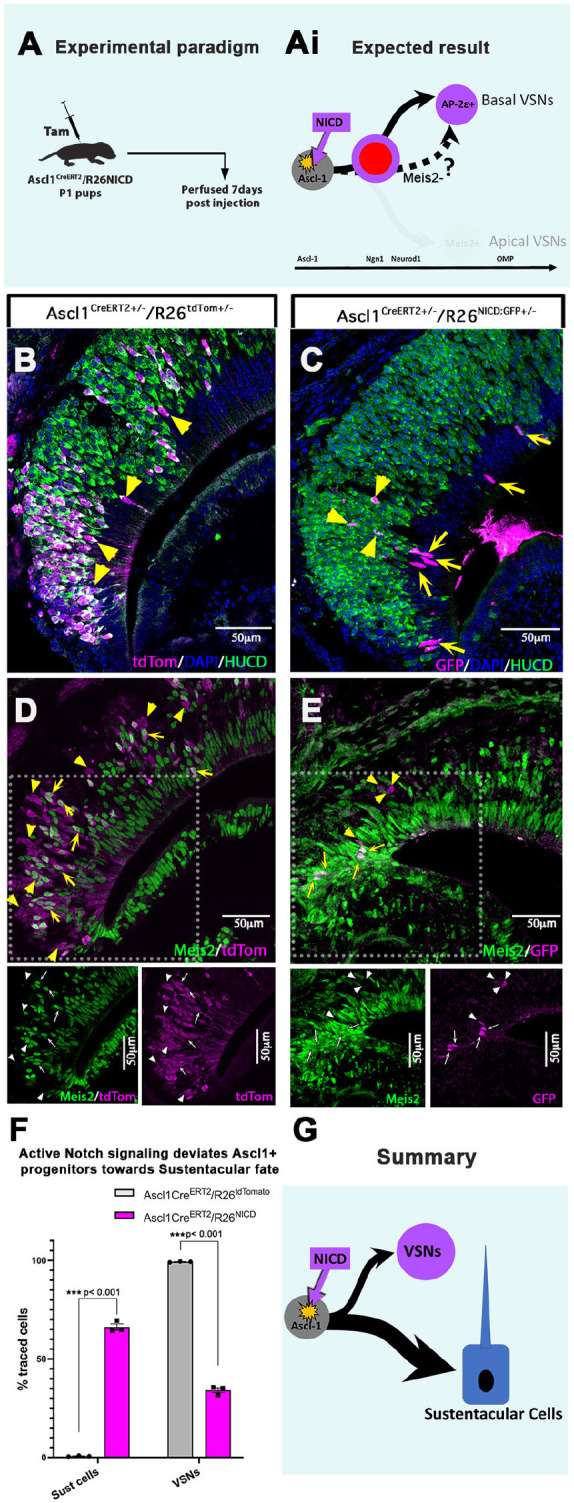
**Ectopic expression of NICD at the Ascl1^+^ stage diverts the progenitors towards sustentacular cell fate.** (A) Ascl1Cre^ERT2^/R26NICD and Ascl1Cre^ERT2^/R26tdTom pups were injected with tamoxifen at P1 and perfused at 7 dpi. (Ai) Expected result with NICD overexpression at the Ascl1^+^ stage driving progenitors towards the basal VSN fate. (B,C) Immunofluorescence of tdTom/HUCD in control and GFP/HUCD in NICD-inducible mice at 7 dpi. Arrows highlight NICD^+^/HUCD^−^ sustentacular cells, whereas arrowheads highlight NICD^+^/HUCD^+^ VSNs. (D,E) Immunofluorescence of tdTom/Meis2 in control and GFP/Meis2 in NICD-inducible mice at 7 dpi. Arrows highlight traced Meis2^+^ apical VSNs in control mice and Meis2^+^ sustentacular cells in inducible mice. Arrowheads highlight Meis2^−^ basal VSNs in both control and inducible mice. (F) Quantification of percentage traced tdTom^+^ cells or GFP^+^ cells that are sustentacular cells or VSNs in control and NICD mice, respectively. (G) Summary showing NICD overexpression at Ascl1^+^ stage leads primarily to differentiation of progenitors into non-neuronal sustentacular cells. Statistical analysis based on arcsine-transformed values of the percentage data; unpaired two-tailed *t*-test; *n*=3 biological replicates. Data shown as mean±s.e.m. At 7 dpi, the total number of recombined cells considered for percentage analysis in the control group was 947.3±40.4 and in the mutant group was 90±7.

We induced Cre recombination at P1 in both control Ascl1Cre^ERT2^/R26tdTom^+/−^ and Notch inducible Ascl1Cre^ERT2^/R26NICD^+/−^ pups and assessed cell fate at 7 dpi (Fig. 8A). We performed immunofluorescence against tdTom/HuC/D and GFP/HuC/D to distinguish between sustentacular cells (HuC/D^−^) and VSNs (HuC/D^+^) in control and mutant mice. At 7 dpi in control mice, 99.3±0.1% (mean±s.e.m.) of Ascl1-traced cells became VSNs, and fewer than 1% of the traced cells became non-neuronal sustentacular cells (Fig. 8B,F).

Surprisingly, in Ascl1Cre^ERT2^/R26NICD^+/−^ pups, we found conditional NICD overexpression in Ascl1^+^ progenitors primarily induced the formation of non-neuronal sustentacular cells (Fig. 8C,F). This experiment showed that, in the NICD-inducible pups, only 34.1±1.2% (mean±s.e.m.) of the recombined cells were classified as VSNs (Fig. 8C,F). At 7 dpi, we further analyzed Meis2 expression as it is expressed by sustentacular cells and apical VSNs (Chang and Parrilla, 2016) (Fig. 8D,E). These data showed that the non-neuronal cells formed after ectopic NICD expression became sustentacular cells. All together, these data suggest that sustained activation of NICD in Ascl1^+^ progenitors lead them to differentiate into sustentacular cells rather than into neurons (Fig. 8G).

### Conditional induction of NICD at the Neurog1 precursor stage promotes cells towards basal VSN fate

Based on the intriguing results obtained from the Ascl1^+^ stage, we tested whether later NICD activation, in committed (Neurog1^+^) neuronal precursors, would show a clear role for NICD and Notch signaling in controlling the apical-basal dichotomy. Of note, the Neurog1/Neurod1 stage is coincident to the stage when Notch signaling is more active and when the dichotomy appears to be established (Fig. 2B,D,E,G).

**Fig. 9.**
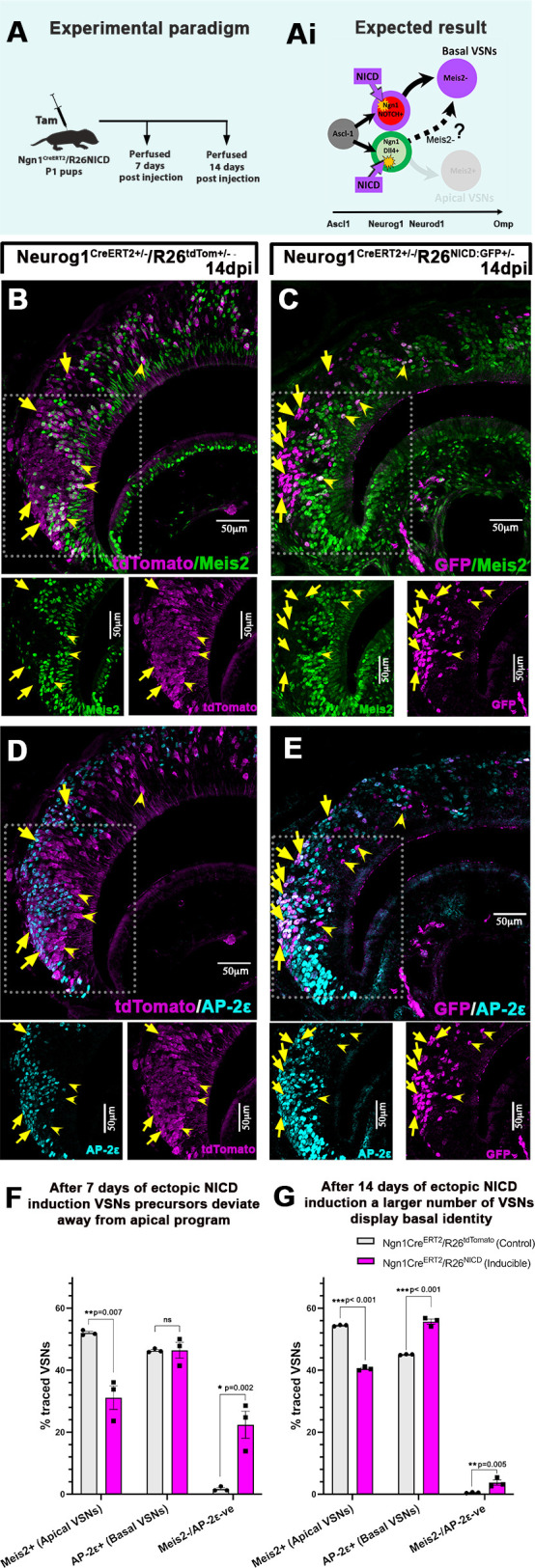
**Ectopic expression of NICD at the Neurog1**^+^ **stage diverts neuronal precursors towards basal VSN fate.** (A) Neurog1Cre^ERT2^/R26tdTom and Neurog1Cre^ERT2^/R26NICD pups were injected with tamoxifen at P1 and perfused at 7 and 14 dpi. (Ai) Expected results show that NICD overexpression at Neurog1^+^ stage may drive progenitors towards the basal VSN fate. (B-E) Immunofluorescence anti tdTom/Meis2/Tfap2e in Neurog1Cre^ERT2^-traced control pups and Neurog1Cre^ERT2^/R26NICD mutant pups at 14 dpi. Panels B and C highlight tdTom/Meis2 staining and panels D and E highlight tdTom/Tfap2e (AP-2ε) staining. Arrows show traced Tfap2e^+^ basal VSNs and arrowheads show Meis2^+^ apical VSNs in both control and inducible mice. (F,G) Quantification of percentage traced tdTom^+^ cells or GFP^+^ cells at 7 dpi and 14 dpi that are Meis2^+^, Tfap2e^+^ VSNs or Meis2/Tfap2e double negative cells in control and NICD mice. Statistical analysis based on arcsine-transformed values of the percentage data; unpaired two-tailed *t*-test; *n*=3 biological replicates. Data shown as mean±s.e.m. At 7 dpi, the average number of recombined cells in the control group was 368.1±23.8 and in the mutant group it was 128.3±35.2. At 14 dpi, the average number of recombined cells in the control group was 509±46.6 and in the mutant group it was 148.5±20.7.

We induced Cre recombination at P1 in both control Neurog1Cre^ERT2^/R26tdTom^+/−^ and Notch inducible Neurog1Cre^ERT2^/R26NICD^+/−^ pups and analyzed cell fate 7 dpi (Fig. 9A). We counted tdTomato^+^ cells and NICD:EGFP^+^ cells to follow the recombined cells in control and inducible R26NICD mice, respectively. In control Neurog1 traced pups, Meis2/tdTom scoring at 7 dpi showed that ∼52±0.4% of the cells expressed the apical VSN marker (Meis2^+^), 46.3±0.3% of the cells are Tfap2e^+^ basal VSNs, and the rest of the population (1.6±0.4%) did not yet express Meis2/Tfap2e (mean±s.e.m.) (Fig. 9F).

In contrast, in the Neurog1Cre^ERT2^/R26NICD inducible mice, 1 week after constitutive NICD overexpression we found that the Meis2^+^ apical VSN population significantly decreased to 31.1±3.8%, whereas Meis2/Tfap2e double negative cells significantly increased to 22.4±4.3%, with the rest of the population being Tfap2e^+^ (mean±s.e.m.) (Fig. 9F).

To further understand whether the NICD-induced Meis2/Tfap2e double negative cells can arrive to express the basal maturation marker Tfap2e (Lin et al., 2018) (Fig. 2C), we also analyzed control and NICD inducible mutants 14 days after tamoxifen injection (Fig. 9A). Corroborating the 7 dpi results, Neurog1Cre^ERT2^/R26NICD mutants at 14 days also showed significant decrease in the Meis2^+^/GFP^+^ population (Fig. 9B-E,G). However, at this stage there is a significant increase in the Tfap2e^+^/basal VSNs from 45±0.08% in control pups to 55.7±0.8% in the mutants, with the rest of the traced cells being Meis2/Tfap2e double negative (mean±s.e.m.) (Fig. 9G). These data suggest that induction of Notch signaling at the Neurog1 stage is sufficient to promote the VSNs towards the basal differentiation program.

## DISCUSSION

The interaction of extrinsic and intrinsic regulators is important for neural stem cells and progenitors to make cell fate decisions and give rise to specific neuronal cell types. The VSE of mice is composed of V1R^+^/Gαi2^+^ apical and V2R^+^/Gαo^+^ basal VSN sub types. Correct development and function of these two main types of VSNs is important for social, sexual, maternal and predator avoidance behaviors of rodents (Chamero et al., 2011; Oboti et al., 2014; Trouillet et al., 2019; Pallé et al., 2020). However, Bcl11b is the only known intrinsic regulator previously shown to affect the initial cell fate decision in the VNO (Enomoto et al., 2011). In the current study, we identified the role of active Notch1 signaling in the cell fate specification of apical and basal VSNs. In the context of T cell development, *Bcl11b* was previously reported to be a direct downstream Notch signaling target gene (Li et al., 2010). Our results confirm a similar Notch-Bcl11b activation axis in the VNO.

In this study, scRNA-seq analysis of adult mouse VNO at the neuronal differentiation dichotomy identified specific expression of *Dll4* ligand and *Notch1* receptor in the apical and basal committed neuronal precursors, respectively. Notably, other studies have previously highlighted the expression of *Notch1* and *Dll4* in the VNO at different developmental stages. In particular, broad *Notch1* and *Dll4* expression have been reported throughout the developing VNO at embryonic stages (Benedito and Duarte, 2005; Wakabayashi and Ichikawa, 2007). However, in postnatal stages, similar to our observations, restricted expression of *Notch1* has been previously reported in the marginal zones, the neurogenic areas of the VNO (Benedito and Duarte, 2005; Wakabayashi and Ichikawa, 2007). These data suggested to us to investigate potential roles for Notch-Delta signaling in establishing VSN dichotomy.

Notch is an evolutionarily conserved juxtacrine signaling pathway associated with inhibitory interactions that can determine the cell fates adopted by juxtaposed differentiating cells (Collier et al., 1996; Artavanis-Tsakonas et al., 1999). Notch inhibitory ligand-receptor interactions rely on non-symmetric expression of a Notch transmembrane receptor and a Notch ligand on neighboring cells. Notch activation in one cell prevents it from assuming the same fate as the neighboring cell expressing the ligand. Notch paralogs (Notch1, Notch2, Notch3, Notch4), three delta-like ligands (Dll1, Dll3, Dll4) and two jagged-like ligands (Jag1, Jag2) have been identified in mammals (Bray, 2016). In this study, we performed loss-of-function and gain-of-function experiments at the Ascl1^+^ neuronal progenitor stage and Neurog1^+^ neuronal precursor stage to probe the necessity and sufficiency of Notch signaling in establishing neuronal diversity in the VNO. *Notch1* receptor knockout from the Ascl1^+^ neuronal progenitor stage onwards (Ascl1Cre^ERT2^ driver) caused most of the developing neurons to enter the apical/Meis2^+^ differentiation program. This suggests that neuronal progenitors will default towards apical VSN fate if not instructed otherwise. Vomeronasal precursors with active Notch signaling go on to express *Bcl11b* (Enomoto et al., 2011) and then *Tfap2e* at a later maturation stage (Lin et al., 2018). Notably, Notch signaling can occur in both canonical and non-canonical fashions (Siebel and Lendahl, 2017). By performing *Rbpj* conditional ablation, a key transcription factor in the canonical pathway (Contreras-Cornejo et al., 2016), we showed that the activation of the basal VSN program relies on canonical Notch signaling (Figs 5 and 6).

Activation of signaling pathways at different stages of development can lead to different cellular outcomes. By using the Neurog1Cre^ERT2^ driver, rather than the Ascl1Cre^ERT2^, we were able to ablate Notch1 expression at later stages of VSN neuronal differentiation. Interestingly, *Notch1* ablation after Cre recombination induction at Neurog1 stage was no longer effective at altering the differentiation trajectories of the VSNs (Fig. S5). Based on this, we propose that, in the developing VSNs, Notch activation has to occur within a crucial developmental time window during which cells still retain sufficient cellular plasticity.

To understand whether the differences in the VSN cell types observed after Notch signaling ablation were due to aberrant proliferation or cell death, we quantified these two variables (Fig. 7). Our data showed no differences in cell division or apoptosis. However, we found that 3 days after recombination, most of the proliferative cells were negative for Bcl11b, which is a known Notch signaling target with a role in directing basal VSN differentiation (Li et al., 2010; Enomoto et al., 2011). These data confirm an early role of canonical Notch signaling in directing the expression of alternative differentiation programs starting from proliferative precursors in the VNO.

Ectopic activation of Notch signaling has been previously shown to be able to alter cell fate (Jadhav et al., 2006). Consistently, our conditional ectopic NICD gain-of-function experiments in the VNO led to distinct phenotypes depending on the timing of NICD induction. NICD activation in Ascl1^+^ neural committed progenitors, which normally give rise to VSNs (see Fig. 8B), appears to be sufficient to redirect their differentiation towards sustentacular cell fate. However, owing to the undetectability of GFP in the early stages (1 or 2 dpi), we could not assess whether early changes in proliferation or cell death compromised the formation of neurons from the recombined cells. The non-neuronal fate may result from NICD-mediated negative regulation of neurogenic transcription factors (Kunnimalaiyaan et al., 2006; Imayoshi et al., 2013) and by the activation of the alternative sustentacular differentiation program. Notably, it has been shown that NICD overexpression in both multipotent horizontal basal cells (HBCs) and globose basal cells of the main olfactory epithelium is sufficient to induce sustentacular cell differentiation (Herrick et al., 2018). Moreover, our scRNA-seq data also identified the expression of downstream Notch targets in the VNO sustentacular cell population, suggesting a role for Notch signaling in their formation (Fig. S7). Although, Ascl1^+^ progenitors have more restricted potency than HBCs (Fletcher et al., 2017), the phenotypic change of vomeronasal Ascl1^+^ progenitors in response to NICD implies that, even at this stage, the chromatin landscape is still plastic enough to be rearranged by active Notch signaling (Aydin et al., 2019; Rahe and Hobert, 2019).

On the other hand, NICD activation at the Neurog1^+^ stage did not convert neuronal cells to sustentacular cells, but rather increased the proportion of basal VSNs. This suggests that, at the Neurog1^+^ stage, VSN precursors have reached a level of commitment sufficient to prevent them from being reprogrammed to sustentacular cells (Rahe and Hobert, 2019). However, at Neurog1^+^ stage, we observed that active Notch signaling is sufficient to divert VSN precursors towards the basal VSN fate. After Neurog1 Cre-mediated NICD induction, we also observed that several VSNs retained the apical identity (Fig. 9F,G). Notably, Neurog1 expression starts immediately after Ascl1, and it is maintained until the apical-basal VSN dichotomy is established (see Fig. 2B). Therefore, Neurog1Cre recombination, and NICD induction, can occur at different stages before or after the differentiation dichotomy occurs. It is therefore possible that the plasticity and the ability of Notch signaling to reprogram VSNs to basal fate is limited to a short developmental window (Rahe and Hobert, 2019).

Evolution of new sensory neuronal types in animals can have an important role in determining their social and environmental fitness by expanding their ability to detect, compute and respond to new stimuli. Vomeronasal receptor types V1R and V2R are functionally and evolutionarily unrelated superfamilies of receptors, and previous studies have highlighted the diversity of V1R^+^ and V2R^+^ VSN populations across many vertebrate species (Grus and Zhang, 2006; Shi and Zhang, 2007). In most mammals (e.g. horse, goat, musk shrew, common marmoset, dog and cow), few to no V2R genes appear to be expressed. Notably, rodents and opossum have a strikingly expanded repertoire of functional V2R genes and, in these animals, specialized VSNs segregate into V1R^+^/Gαi2^+^ apical and V2R^+^/Gαo^+^ basal population (Takigami et al., 2000, 2004). In conclusion, our study has revealed a crucial role for Notch signaling in determining the formation of the two main neuronal cell types of the VNO of mice. Our data suggest an important evolutionary role for Notch signaling in driving the cellular diversity of neuroepithelia of vertebrates.

## MATERIALS AND METHODS

### Mouse lines

We purchased Ascl1Cre^ERT2^ [*Ascl1^tm1.1(Cre/ERT2)Jejo^*/J, 012882], Neurog1Cre^ERT2^ [B6;129P-Tg(Neurog1-cre/ERT2)1Good/J, 008529], Notch1^fl/fl^(B6.129X1-*Notch1^tm2Rko^*/GridJ, 007181), Rbpj^fl/fl^ (C57BL/6J-*Rbpj^em2Lutzy^*/J, 034200), R26NICD [*Gt(ROSA)26Sor^tm1(Notch1)Dam^*/J, 008159], R26tdTom [B6.Cg-*Gt(ROSA)26Sortm9^(CAG-tdTomato)Hze^*/J, 007909] mouse lines from The Jackson Laboratory. The *Foxn4^+/lacZ^* mouse line was previously generated (Li et al., 2004). Genotyping was conducted following the suggested primers and protocols from The Jackson Laboratory. Mice of either sex were used for immunohistochemistry and immunofluorescence experiments. All experiments involving mice were approved by the University at Albany Institutional Animal Care and Use Committee (IACUC).

### scRNA-seq

The VNOs of P60 C57BL/6J male mice were isolated and dissociated into single cell suspensions using neural isolation enzyme/papain (Thermo Fisher Scientific, 88285) in Neurobasal Medium (Thermo Fisher Scientific, 21103049) with 0.5 mg/ml Collagenase A, 1.5 mM L-cysteine and 100 U/ml DNAse I incubated at 37°C. The dissociated cells were then washed with Hank's Balanced Salt Solution (HBSS) and reconstituted in cell freezing medium (90% fetal bovine serum, Thermo Fisher Scientific, 26140079; 10% DMSO, Sigma-Aldrich, 472301). Cells were frozen from room temperature to −80°C at a freeze rate of −1°C/min. The single cell suspension was sent to SingulOmics for high-throughput single cell gene expression profiling using the 10x Genomics Chromium Platform.

Single cell library preparation and sequencing were conducted by SingulOmics. Cryopreserved viable single cell suspensions were thawed, washed, resuspended in cell culture media with 0.04% bovine serum albumin and counted. Viable cell suspensions were then loaded into the Chromium Controller (10x Genomics) to generate gel beads-in-emulsion (GEM), with each GEM containing a single cell as well as barcoded oligonucleotides. We made two samples targeting 10,000 cells to be captured per sample. Next, the GEMs were placed in the SimpliAmp 96-well Thermal Cycler (Thermo Fisher Scientific) and reverse transcription was performed in each GEM (GEM-RT). After the reaction, the complementary cDNA was amplified and cleaned using Silane DynaBeads (10x Genomics) and the SPRI select Reagent kit (Beckman Coulter). Amplified full-length cDNAs from poly-adenylated mRNA were then used to generate a 3′ Gene Expression library (Chromium Next GEM 3′ Single Cell Reagent kits v3.1, dual index) following the manufacturer's instructions (10x Genomics). Amplified cDNAs and the libraries were measured using Qubit dsDNA HS assay (Thermo Fisher Scientific) and quality assessed using BioAnalyzer (Agilent Technologies). Libraries were sequenced on a NovaSeq 6000 instrument (Illumina), and fastq files of two samples were generated using Illumina bcl2fastq. Fastq files were subsequently processed using 10x Genomics Cell Ranger analytical pipeline (v4.0.0) and mouse mm10 reference. Cellranger aggr was used to aggregate outputs from both the libraries and the aggregated filtered processed files were finally taken as input to do quality control check and further downstream clustering analysis using Seurat 3.2.3. scRNA-seq data from OmpCreHet P10 animals has been generated as described above.

### Tamoxifen treatment

Tamoxifen (Sigma-Aldrich, 10540-29-1), was dissolved in corn oil at 20 mg/ml concentration. For all tamoxifen-inducible experiments we injected tamoxifen once intraperitoneally at P1 at a dose of 80 mg/kg body weight and perfused at indicated postnatal days.

### Tissue preparation

Collected tissues were perfused with PBS followed by 3.7% formaldehyde in PBS. Noses were immersion fixed in 3.7% formaldehyde in PBS at 4°C for 1-2 h depending on mouse age. All samples were cryoprotected in 30% sucrose in PBS overnight at 4°C then embedded in Tissue-Tek O.C.T. Compound (VWR, 25608-930) using dry ice and stored at −80°C. Tissue was cryosectioned using a CM3050S Leica cryostat at 16 µm for VNOs and collected on VWR Superfrost Plus Micro Slides (Radnor) for immunostaining. All slides were stored at −80°C until ready for staining. We included both males and females in our study but did not make any sex distinction for the analyses.

### Immunofluorescence

Citrate buffer (pH 6.0) antigen retrieval was performed (Lin et al., 2018), for all the antibodies indicated with an asterisk. Primary antibodies and concentrations used in this study were: goat anti-AP-2ε (*with antigen retrieval 1:500 and without antigen retrieval 1:200, AF5060, R&D Systems), chicken anti-GFP (1:3000, ab13970, Abcam), rabbit anti-GFP (1:1000, A-6455, Molecular Probes), *rabbit anti-Ki67 (1:1000, D3B5, Cell Signaling Technology), *mouse anti-Ki67 (1:500, 9449, Cell Signaling Technology), *mouse anti-Meis2 (1:500, sc-515470, Santa Cruz Biotechnology), rabbit anti-Meis2 (*with antigen retrieval 1:1000 and without antigen retrieval 1:500, ab73164, Abcam), *mouse anti-NeuroD1 (1:100, sc-46684, Santa Cruz Biotechnology), *goat anti-NeuroD1 (1:500, AF2746, R&D Systems), *rabbit anti-Notch1 (1:50, D1E11, Cell Signaling Technology), goat anti-Dll4 (*with antigen retrieval 1:50 and without antigen retrieval 1:25, AF1389, R&D Systems), *rabbit anti-NICD activated (1:75, D3B8, Cell Signaling Technology), *mouse anti-DsRd (1:500, TA180084, Origene), *rabbit anti-DsRd (1:500, 600-401-379, Rockland), HuC/D 8 μg/ml (Molecular Probes), rabbit anti-Foxn4 (1:50; Li et al., 2004), *rabbit anti-Gαo1 (1:1000, PA5-59337, Invitrogen), *mouse anti-Gαi2 (1:250, clone L5, MAB3077, Millipore).

Species-appropriate secondary antibodies conjugated with either Alexa Fluor 488, Alexa Fluor 594, Alexa Fluor 568 or Alexa Fluor 680 plus were used for immunofluorescence detection (Molecular Probes and Jackson ImmunoResearch Laboratories). Sections were counterstained with 4′,6′-diamidino-2-phenylindole (DAPI) (1:3000; CalBiochem, 268298) and coverslips were mounted with FluoroGel (Electron Microscopy Services, 17985-11). Confocal microscopy pictures were taken on a Zeiss LSM 710 microscope. Epifluorescence pictures were taken on a Leica DM4000 B LED fluorescence microscope equipped with a Leica DFC310 FX camera. Images were further analyzed using FIJI/ImageJ software.

### scRNA-seq quality control and cell clustering

Quality control (QC), and clustering and downstream analysis was performed using Seurat [3.2.3] package in R. Basic filtering was carried out in which all genes that were expressed in ≥3 cells and all cells with at least 200 detected genes were included. QC was based on number of genes and percentage of mitochondrial genes – all cells that expressed >9000 genes and >5% mitochondrial genes were not included in the analysis. After filtering, 10,582 cells were included for the clustering and analysis. Top 2000 highly variable genes across the population were selected to perform principal component analysis (PCA) and the first 30 principal components were used for cell clustering, which was then visualized using UMAP. Stem cells, neuronal progenitors, precursors and immature neuronal cell types were identified based on the expression of known genes. These cell types were specifically chosen to subset, and the top 2000 highly variable genes and top 15 principal components were used to cluster and create new Seurat object 2. Similarly, Seurat object 3 was created by focusing on clusters only at VSN dichotomy and the top 20 principal components were used to cluster and visualize. All downstream analysis that identified Notch1-Dll4 signaling was carried out using Seurat object 3.

For the integrated P10 and P60 scRNA-seq analysis, we preprocessed and determined highly variable features in P10 and P60 data separately. Seurat integration method was used to identify integration anchors, integrate both datasets and the first 35 principal components were used for downstream cell clustering.

### Pseudotime analysis of cell population

To further confirm the VSN dichotomy, we used Monocle3 to perform pseudotime analysis, where Ascl1^+^ cells were chosen as root node. We used Seurat wrappers package to directly convert Seurat object 2 into cell dataset format.

### Experimental design, quantification and statistical analyses of microscopy data

All data were collected from mice kept under similar housing conditions, in transparent cages on a normal 12 h light/dark cycle. Tissue collected from either males or females in the same genotype/treatment group was analyzed together unless otherwise stated; ages analyzed are indicated in text and figures. Measurements of VSE and cell counts were performed on confocal images of coronal serial sections immunostained for the indicated targets. Measurements and cell counts were carried out using ImageJ. The data are presented as mean±s.e.m. unless otherwise specified. Prism 9.0.1 was used for statistical analyses, including calculation of mean values and s.e.m. Values of traced cells in distinct genetic backgrounds were compared as percentage traced. Percentage values were transformed into Arcsine values. *P*-values were calculated using unpaired two-tailed *t*-test using the arcsine transformed values: *P<0.05, **P<0.01, ***P<0.001, ns, not significant. Each animal is considered as a biological replicate and both males and females were included in the loss-of-function and gain-of-function studies. Sample sizes and *P*-values are indicated as single points in each graph and/or in figure legends.

## Supplementary Material

Supplementary information

Reviewer comments
