# Supplementary material for: Notch signaling determines cell-fate specification of the two main types of vomeronasal neurons of rodents
Source: Development. 2022 Jul 4;149(13):dev200448. doi: 10.1242/dev.200448 (PMC9340558; doi:10.1242/dev.200448)
Supplement: Supplementary information [file develop-149-200448-s1.pdf]

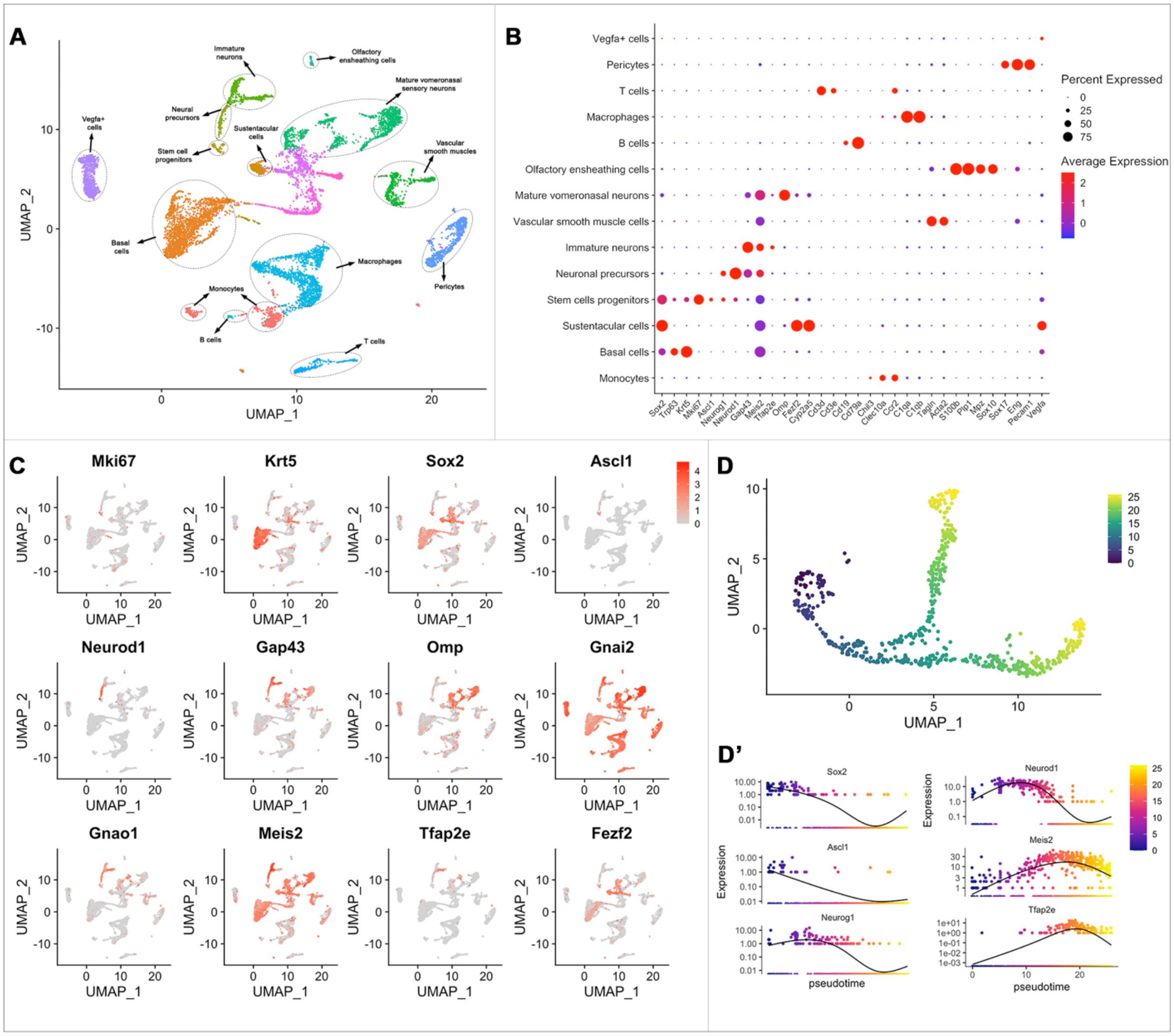

Fig. S1. scRNA-seq from p60 mice identifies different cell types in the adult mouse VNO. **A)** UMAP dimensional reduction plot of Seurat object 1 shows neuronal and non-neuronal cell clusters of the VNO. Each color corresponds to a cluster of cells that have similar transcriptomic profile. **B)** Dot plot visualization of all the genes used to identify cell types in the scRNA-seq data set. Unidentified clusters are not shown here **C)** Feature plots of various genes related to stem cell progenitors, precursors, immature and mature VSNs and sustentacular cells in Seurat object1. **D)** The single cell pseudotime trajectory of Seurat object2 predicted by Monocle Seurat wrapper and visualized by UMAP. Cells are ordered in pseudotime by choosing Ascl1+ cells as the root node and colored in a gradient from purple to yellow. **D')** Dynamic expression of small set of genes as a function of pseudotime.

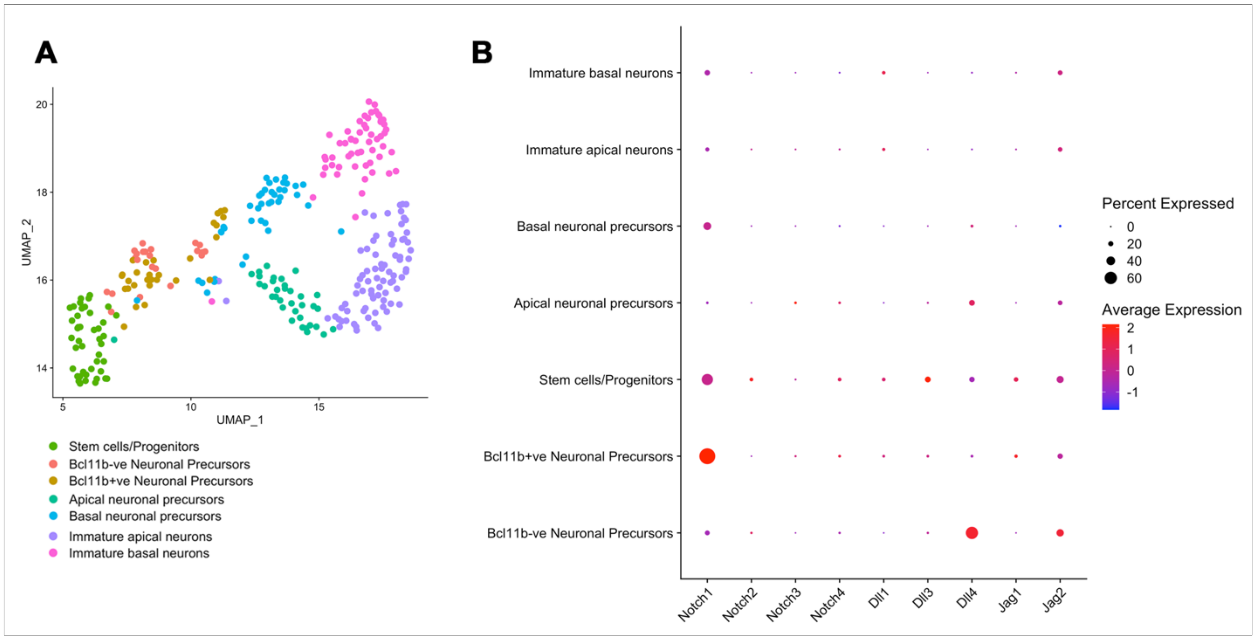

Fig. S2. Notch1 and Dll4 is the highly expressed receptor and ligand combination at the VSN dichotomy. A) UMAP dimension plot of Seurat object 3 from p60 scRNA-seq specifically focusing on the VSN apical-basal split. **B)** Dot plot visualization of all Notch receptors and ligands gene expression in the Seurat object3 highlighting Notch1 and Dll4 expression in Bcl11b+ vs Bcl11b- precursors.

Development • Supplementary information

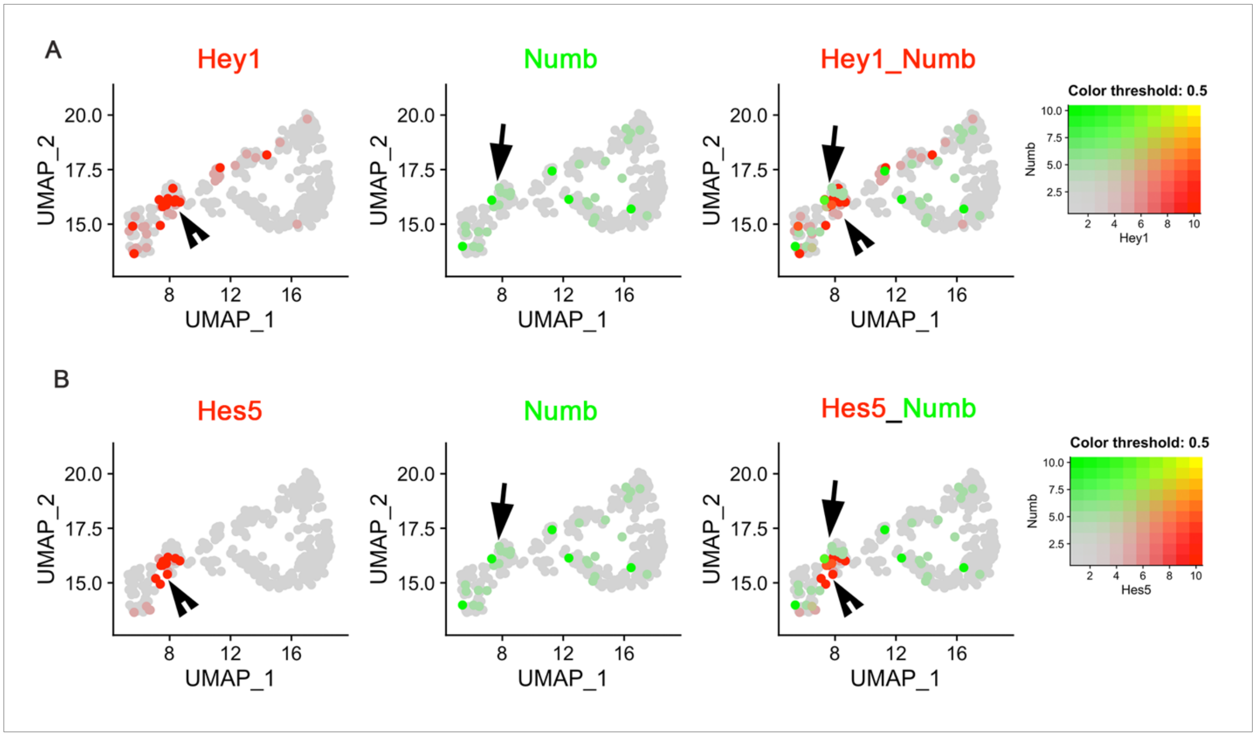

Fig. S3. Expression of Numb and downstream Notch targets are mutually exclusive at the VSN dichotomy. Blended feature plots of Notch target genes Hey1 and, Hes5 vs Numb, which is a negative Notch regulator, from p60 scRNA-seq shows that Notch target genes are not expressed in the same populations as Numb at the VSN dichotomy.

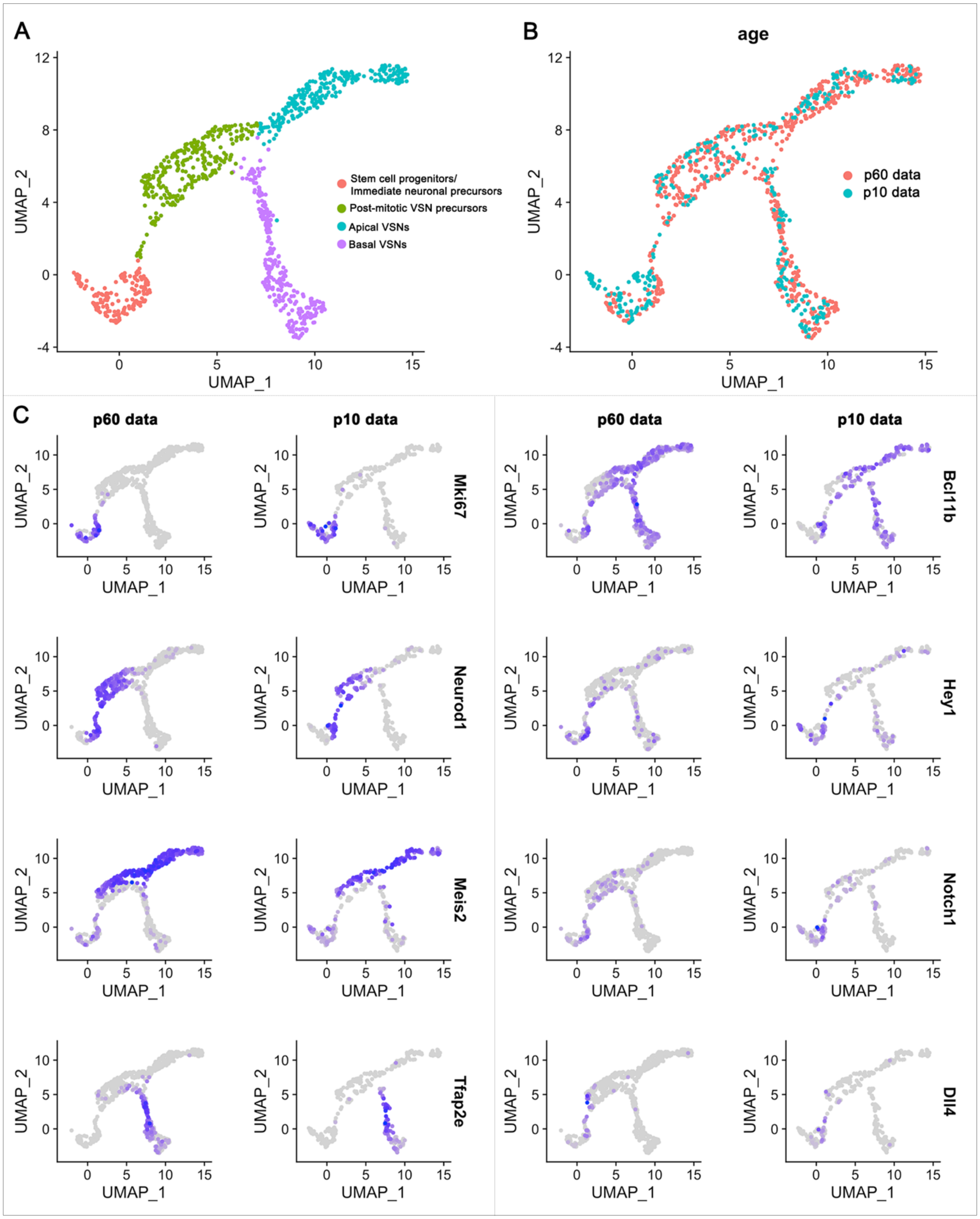

Fig. S4. Integrated scRNA-seq data from P60 and P10 mice shows conserved developmental trajectory across ages. A) UMAP projection of integrated scRNA-seq data from P60 and P10 ages. Each color corresponds to specific cell type **B)** Integrated

Development • Supplementary information

UMAP projections of P10 (teal) and P60 (orange) data grouped by age shows overlapping trajectories. **C)** Feature plots of key developmental markers including Notch signaling pathway genes (Bcl11b, Hey1, Notch1, Dll4) at P60 and P10 shows similar expression pattern across the developmental trajectories.

Development • Supplementary information

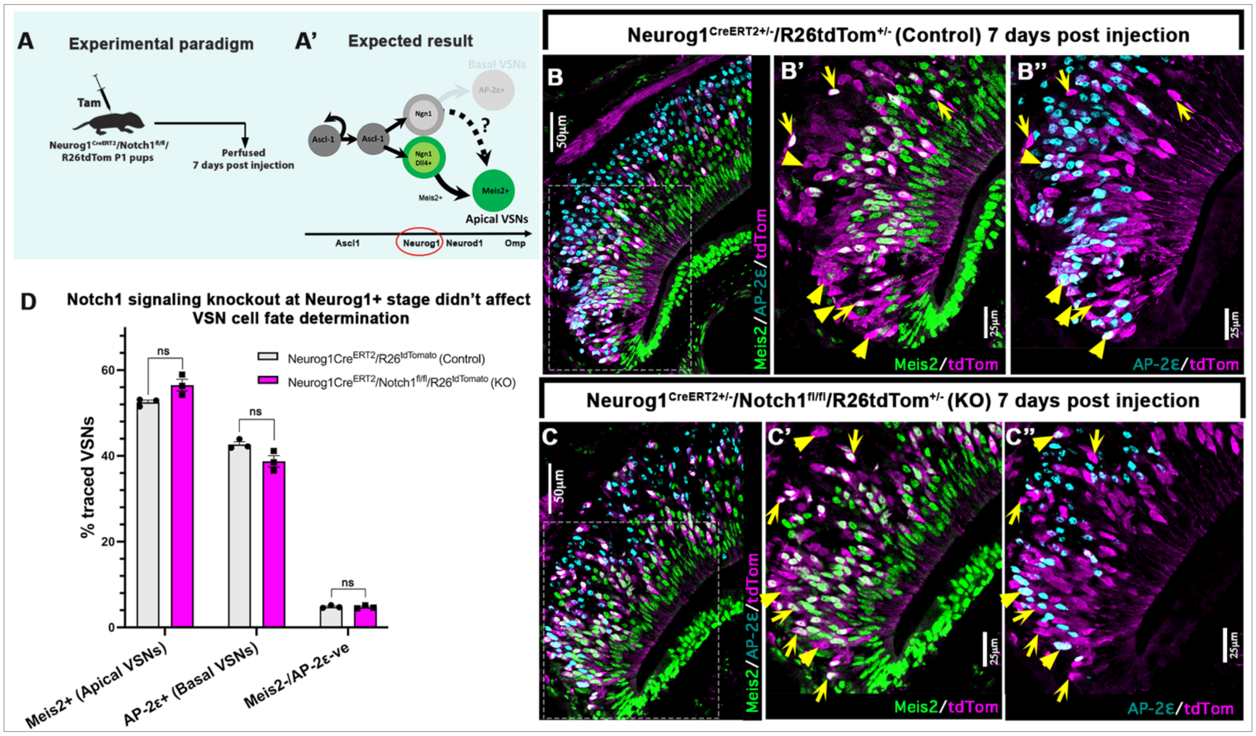

Fig. S5. Notch signaling knockout induced at Neurog1 stage didn't affect apical-basal VSN differentiation. A) Cartoon summarizing experimental design of Notch1 loss of function study at Neurog1 stage. Neurog1Cre^{ERT2}/Notch1$^{fl/fl}$/R26tdTom and Neurog1Cre^{ERT2}/R26tdTom pups were injected with tamoxifen at the P1 stage and perfused at 7dpi. **A')** The cartoon depicts expected results with Notch1 knockout at Neurog1 stage driving the progenitors towards the apical VSN fate. Red circle highlights Neurog1 stage where recombination is induced. **B)** Triple immunofluorescence of Meis2, AP-2ε and tdTom in Neurog1Cre^{ERT2} induced control pups at 7dpi. **B', B'')** Magnification of the box in image B showing Meis2/tdTom and AP-2ε/tdTom double immunofluorescence images respectively. Arrows highlight traced neurons that are Meis2+ apical VSNs and arrowheads highlight traced neurons that are AP-2ε+ basal VSNs. **C)** Triple immunofluorescence of Meis2, AP-2ε and tdTom in Neurog1Cre^{ERT2} induced Notch1 conditional KO pups at 7dpi. **C', C'')** Magnification of the box in image C showing Meis2/tdTom and AP-2ε/tdTom double immunofluorescence images respectively. Arrows highlight traced neurons that are Meis2+ apical VSNs and arrowheads highlight traced neurons that are AP-2ε+ basal VSNs. **D)** Quantification of the percentage traced VSNs that are Meis2+ apical, AP-2ε+ basal VSNs and Meis2/AP-

2ε double negative cells in Neurog1Cre^{ERT2} induced control (gray) and Notch1 KO mice (magenta) at 7dpi stages. Values of traced cells in distinct genetic backgrounds were compared as %. Percentage values were transformed into Arcsine values. P values were calculated using unpaired two-tailed Student's *t*-test using the arcsine transformed values; n=3 biological replicates. Both males and females were included in the analysis. Data shown as mean±SEM; *$P<0.05$, **$P<0.01$, ***$P<0.001$. ns- not significant. At 7dpi, the average number of tdTom+ cells in control group: 360.9±12.3; in cKO: 414.45±26.5.

Development • Supplementary information

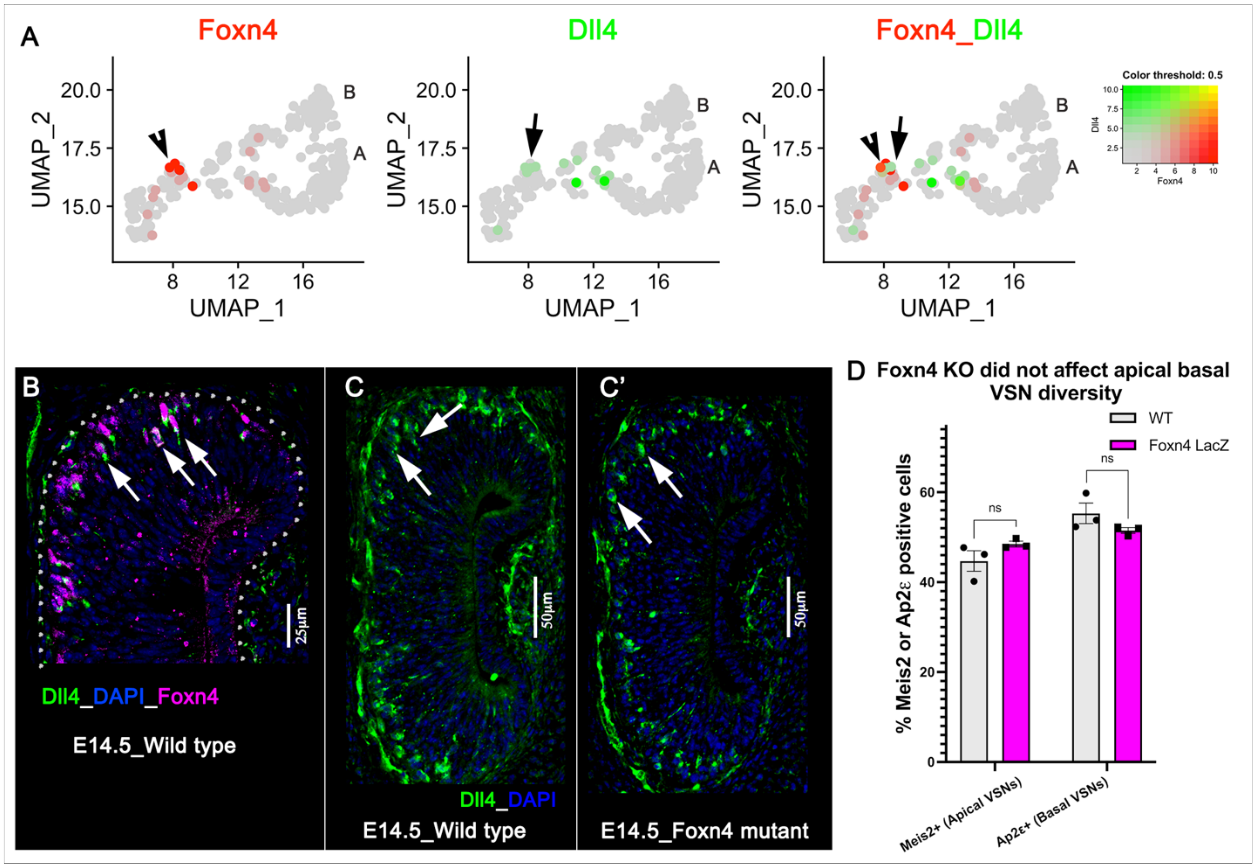

Fig. S6. Foxn4 is redundant in inducing Dll4 expression A) Blended feature plots of Foxn4 and Dll4 from p60 scRNA-seq data show that Foxn4 (arrowhead) is expressed at the apical-basal split along with Dll4 (arrow). **B)** Double immunofluorescence of Dll4 and Foxn4 in wild-type VNO at embryonic day 14.5 (E14.5) shows their colocalization (arrows). **C, C')** Immunofluorescence of Dll4 in wild-type and Foxn4 mutant VNOs show detectable Dll4 expression (arrows). **D** Quantification of % Meis2-positive and AP-2ε-positive cells in wild-type and Foxn4 mutant VNOs at E14.5. Percentage values were transformed into Arcsine values. P values were calculated using unpaired two-tailed Student's *t*-test using the arcsine transformed values; n=3 biological replicates. Data shown as mean±SEM; *P<0.05, **P<0.01, ***P<0.001. ns- not significant. The average number of Meis2+ and AP-2ε+ cells in control group are 89.2±4.6 and 110.3±4.4 respectively; in mutant Meis2+ and AP-2ε+ cells are 91.2±8.6 and 96.7±9.6 respectively.

Development • Supplementary information

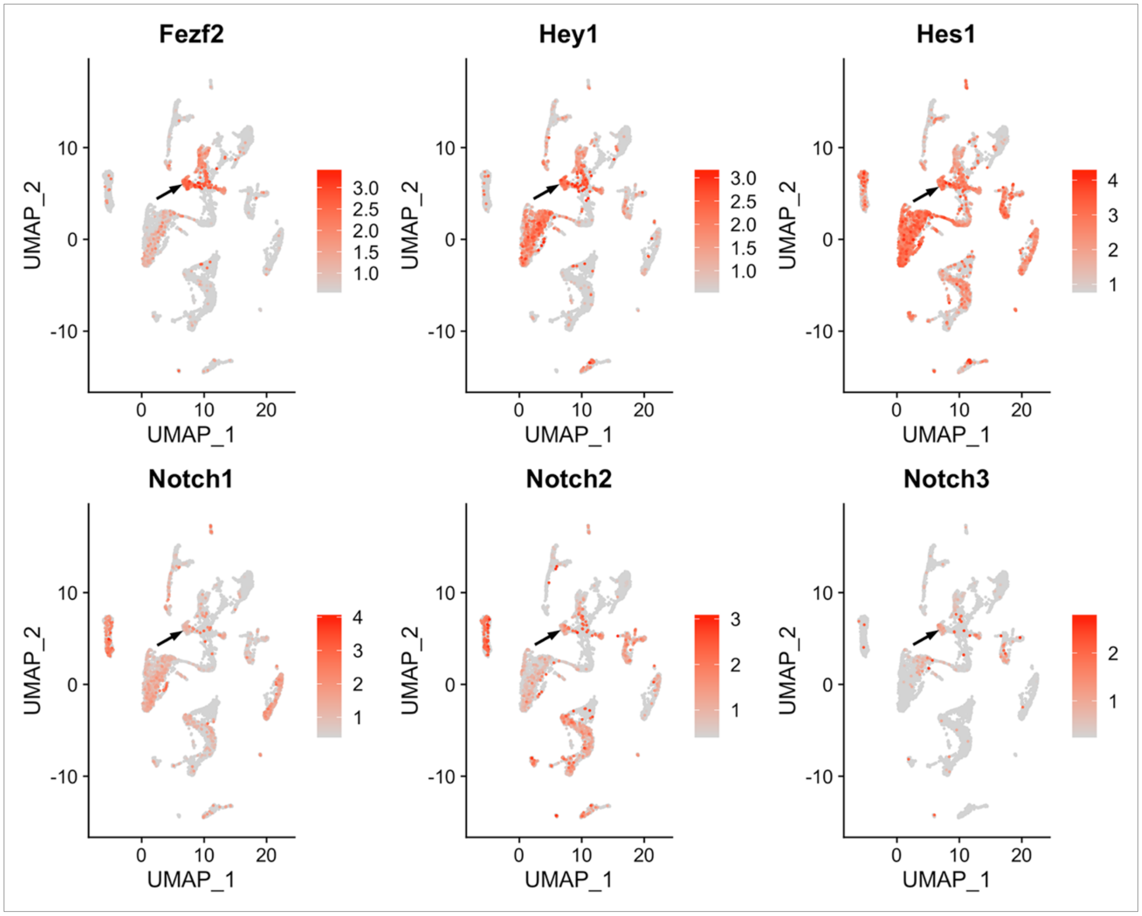

Fig. S7. scRNA-seq analysis from p60 mice reveals the expression of downstream Notch targets in sustentacular cells. A) Feature plot of Fezf2 identifies Sustentacular cells in the Seurat object1. **B, C)** Feature plots of downstream Notch signaling target genes Hey1 and Hes1 show their expression in the Sustentacular cell cluster. **D, E, F)** Feature plots of Notch signaling receptors Notch1, Notch2 and Notch3 show their expression in the Sustentacular cell cluster. Arrow highlights sustentacular cell cluster in all feature plots.
