# Supplementary material for: Notch signaling determines cell-fate specification of the two main types of vomeronasal neurons of rodents
Source: Development. 2022 Jul 4;149(13):dev200448. doi: 10.1242/dev.200448 (PMC9340558; doi:10.1242/dev.200448)
Supplement: Reviewer comments [file develop-149-200448_review_history.pdf]

Notch signaling determines cell-fate specification of the two main types of vomeronasal neurons of rodents

Raghu Ram Katreddi, Ed Zandro M. Taroc, Sawyer M. Hicks, Jennifer M. Lin, Shuting Liu, Mengqing Xiang and Paolo E. Forni

DOI: 10.1242/dev.200448

Editor: Francois Guillemot

Review timeline

Original submission:	10 December 2021
Editorial decision:	28 January 2022
First revision received:	4 May 2022
Editorial decision:	6 June 2022
Second revision received:	15 June 2022
Accepted:	15 June 2022

Original submission

First decision letter

MS ID#: DEVELOP/2021/200448

MS TITLE: Notch signaling determines cell-fate specification of the two main types of vomeronasal neurons of rodents.

AUTHORS: Raghu Ram Katreddi, Ed Zandro M Taroc, Sawyer M Hicks, Jennifer M Lin, Shuting Liu, Mengqing Xiang, and Paolo E Forni

I have now received the reports of three referees on your manuscript and I have reached a decision. The reports are appended below and you can access them online: please go to BenchPressand click on the 'Manuscripts with Decisions' queue in the Author Area.

As you will see, all the referees are enthusiastic about your work, but they also have significant criticisms and recommend a substantial revision of your manuscript before we can consider publication. In particular, Referee 1 requests that you perform a more extensive characterisation of Ascl1-CreER; Notch1 fl VNO, including investigations of cell death and proliferation, and analysis of mice at earlier and later stage. They also ask that you delete Notch1 using the Neurog1-CreER line, which would complement your NICD overexpression analysis and would strengthen the study, but if the mice have not been generated yet, the data would take too long to produce for the revision and could be omitted. Similarly, the analysis of Rbpj deletion can be omitted. If you are able to revise the manuscript along the lines suggested by the three referees, which may involve further experiments, I will be happy to receive a revised version of the manuscript. Your revised paper will be re-reviewed by the original referees, and its acceptance will depend on your addressing satisfactorily all their major concerns. Please also note that Development will normally permit only one round of major revision.

We are aware that you may be experiencing disruption to the normal running of your lab that make experimental revisions challenging. If it would be helpful, we encourage you to contact us to discuss your revision in greater detail. Please send us a point-by-point response indicating where you are able to address concerns raised (either experimentally or by changes to the text) and

© 2022. Published by The Company of Biologists under the terms of the Creative Commons Attribution License (https://creativecommons.org/licenses/by/4.0/).

where you will not be able to do so within the normal timeframe of a revision. We will then provide further guidance. Please also note that we are happy to extend revision timeframes as necessary.

Please attend to all of the reviewers' comments and ensure that you clearly highlight all changes made in the revised manuscript. Please avoid using 'Tracked changes' in Word files as these are lost in PDF conversion. I should be grateful if you would also provide a point-by-point response detailing how you have dealt with the points raised by the reviewers in the 'Response to Reviewers' box. If you do not agree with any of their criticisms or suggestions please explain clearly why this is so.

Reviewer 1

Advance summary and potential significance to field

In their manuscript, Katreddi et al. address the role of Notch signaling in the generation of vomeronasal sensory neurons. They performed scRNA sequencing of the vomeronasal sensory epithelium and performed a detailed computation analysis of the approx. 10,000 cells in the data set. They focused on the neural progenitor and immature neuronal stages to address fate segregation between apical and basal vomeronasal sensory neurons. Their analysis proposes that Notch signaling regulates the fate of a common Ascl1+ progenitor to V1R and V2R vomeronasal sensory neurons. They performed conditional Notch1 ablation from Ascl1+ cells and conditional gain of function of Notch1 signaling in Ascl1+ and Neurog1+ progenitors. Their results indicate that Notch1 can control the generation of V1R versus V2R (apical and basal) vomeronasal sensory neurons. The bioinformatics data is well presented and clear although more description of the gene expression across all cells would be interesting as they focus on a very minor population here. The genetics data are interesting but need deepening and the mechanisms of action of Notch signaling in the control of vomeronasal sensory neuron fate are not formally show. The finding that Notch controls cell fate in what is claimed to be a committed progenitor is interesting.

Comments for the author

Main concerns The bioinformatic analysis focusses on a small fraction of the cells in the UMAP analysis. It would be good to show the expression of the genes of interest on the entire cell populations shown in Figure 1A. These data can be included in the Supp Figures if needed but it will be interesting to see the distribution of mitotic cells, progenitor markers, neuronal and none neuronal cell population.
The Ascl1-CreER Notch1 LOF experiments should be expanded to look at earlier (1 dpi) and later time points. The analysis should also include cell death analyses and detailed analysis of proliferation. The lineage tracing downstream of Ascl1-CreER is also superficial and should be extended.
It would be good to show confirmatory data showing the distribution of Notch signaling components in the in the early vomeronasal epithelium - for example by in situ hybridization. The Notch1, Delta4 and NeuroD1 immunostaining imply that Notch signaling is restricted to the MZ and does not seem to be equal at all margins (see Figure 3).
The Notch1 LOF should be extended to Neurog1-CreER deletion and analysis of cell fate.
The authors need to present some evidence that the loss of Notch1 on cell fate decisions is due to canonical Notch signaling. Currently, the evidence is rather week. Ideally the authors would perform similar loss of function experiments deleting Rbpj.
The authors claim a reduced recombination of the Rosa-NICD locus to explain the reduced number of recombined cells. This may be true but needs to be controlled with a more extensive analysis of cell proliferation and cell death at early and later stages of development.
The scRNA-seq is a snapshot of gene expression. This is particularly true for the expression of the Notch signaling and proneural genes which are presumably high dynamic and presumably also oscillatory. The predictions of lineage from the bioinformatics are purely predictions without experimental substantiation.
The authors should discuss this in their manuscript.
The author claim a shift in apical to basal vomeronasal sensory neuronal fate due to manipulation of Notch signaling but this seems to be based on the expression of very few markers. The authors should substantiate a change in fate rather than a miss-expression of a few markers.

© 2022. Published by The Company of Biologists under the terms of the Creative Commons Attribution License (https://creativecommons.org/licenses/by/4.0/).

Minor comment.
The authors discuss adult neurogenesis in the introduction but their analysis is purely developmental. This text should be adjusted not to cause confusion.
A better description of the transgenic animals is needed. I suggest to include a scheme of the different alleles in the Supp Figures. This will help the general reader to understand the experimental approach.
Are sustentacular cells a normal progeny of Ascl1+ progenitors? When are these cells normally generated in the vomeronasal sensory epithelium? This should be included in the introduction and expanded in the discussion.
In Figure 2 UMAP plots the authors use color-coded titles for the plots. This would be beneficial in Supp Figures 2, 4 and 5.
The authors need to state the statistical analysis used, SEM or SD and the n-value for all quantifications in the figures. For the numbers shown in the text, the authors need to add Sem or SDs and, when comparing control and mutants state the P-value and test used. In the methods section the authors claimed to have used t-Test for the statistics. Many of the analyses are percentages and t-Test is not the correct analysis unless the data have gone through an Arcsine transformation.
This reviewer does not think that the Ooep data really bring much to this manuscript as there is no experimental relevance presented for the Ooep expression.

Reviewer 2

Advance summary and potential significance to field

This study from Forni and colleagues investigates the mechanisms used to specify apical vs. basal neuronal cell fates in the mouse vomeronasal organ (VNO) using a combination of single cell RNA-sequencing, lineage tracing, and genetic gain- and loss-of-function experiments. Overall this is a lovely study that demonstrates a role of notch-delta signaling in the specification of VNO neuronal types. While interesting in its own right, this is also a nice demonstration of how notch-delta signaling can impact such decisions once the neuronal fate has already been chosen, and not just in neuronal vs. non-neuronal cell fate decisions. I have a few concerns, none that are deal-breakers, that I would encourage the authors to consider before publication.

Comments for the author

On balance the single cell RNA-sequencing analysis looks solid. However by the number of cells in the lineage that are actually included in the main analysis is on the sparse side. I don't think that the authors' conclusions based on these experiments are very far off, given the results of subsequent Ascl1-cre lineage tracing and gain- and loss-of-function experiments. However they might temper their conclusions as an analysis based on deeper sequencing might reveal some nuances.
Similarly, the lineage tracing results with NICD gain-of-function experiments are are a bit marginal to support the conclusions, considering the low efficiency of cre recombination/activation of the NICD allele.
The statistical analysis of lineage tracing and genetic analyses is not clear, or at least not easily accessible. How many mice were used in each figure panel and whether any sex differences were observed. Is an individual mouse consider a "sample" or "n?" Data in the figures are represented as percentages – absolute cell counts would be useful to provide as well. It also appears that the same control data were used for multiple comparisons/figures; if so, this should be stated explicitly (apologies if I missed this).
More information is needed about the cell clusters shown in the UMAP in Figure 1A
– what cell types do the other clusters represent, and based on expression of what marker genes?

Minor comments:
Page 2: typo (subject verb agreement, lines 46-47)
Page 4: How many mice were used? Age range? How many of each sex? The methods indicate P60 male mice. Please provide total number of mice and include that information here (in the main text) as well. Perhaps also add a note whether sex differences would or would not be expected.

© 2022. Published by The Company of Biologists under the terms of the Creative Commons Attribution License (https://creativecommons.org/licenses/by/4.0/).

Page 6 (referring to the Bcl11b results shown in figure 2D vs. 2E-H): The Delta-Notch figures showing inverse expression are more convincing than the Bcl11b result alone, but they are convincing when considered as a whole. My question is whether the Bcl11b result could be supported with other data. There are nice antibodies for Bcl11b IHC that could be used in the GOF and LOF studies, for instance.
Page 11: Typo (line 309)
Page 24 (referring to Figure 1A): A key or color scheme would help in the interpretation of this panel. Do the colors correspond to subclusters within each larger physical cluster? What do the other larger clusters correspond to? (see major concern #2 above)
Page 24 (referring to Figure 1B''): A color key is needed for this heatmap as well.
Page 26 (referring to Figure 3): Perhaps include a directional in the first figure using IHC images (Fig. 3). Do these show a D-V axis or A-P axis?
Page 27 (referring to figure 4): Does the central-peripheral gradient of tdTom expression correspond to a central-peripheral gradient of differentiation/maturation?
Page 28 and onward (referring to bar graphs): Could the authors include the corresponding raw numbers above each bar in 5D and D", ditto for other bar graphs
(Figure 6F and F', Figure S4D)? See also major comment, above.
Page 28 (referring to Figure 5B,C): There seems to be a central-peripheral gradient of the effect of Notch on apical vs. basal fate. If this is the case could the authors comment on it? Are there any known interactions with mitogens/signaling pathways with central-peripheral gradients?
Page 35 (referring to Figure S4): Add scale bars for S4 B-C''

Reviewer 3

Advance summary and potential significance to field

The manuscript by Katreddi et al investigates neuronal diversification in the Vomeronasal Organ (VNO) and describes the effect of manipulations of Notch signaling at stages within the neurogenic cascade. While the role of Notch1 in setting cell fate is certainly not new, the role of Notch in the VNO has not been explored and is important in understanding both VNO development and how Notch is functioning. The authors have very nicely characterized gene expression at discrete stages in the neurogenic cascade and related that to Notch.
The data will be extremely useful for designing future experiments to understand the contextualization of Notch signaling.

Comments for the author

This is a nice set of studies, but there are some issues with their approach and interpretation. The first potential problem, which the authors don't address, is the age disparity between the scRNAseq analysis in adult VNO and their localization and manipulation of Notch pathway components in the neonatal VNO. It is entirely possible that the two situation are not perfectly comparable and some justification needs to be offered. Second, the analysis of the outcome of gene manipulation suffers from 2 potential flaws when trying to assign a role to Notch signaling at a specific point in the cascade. The authors are quite definitive in assigning the role to a specific cell type based on the driver for recombination. There are two potential problems with this interpretation. The rate of decay of the protein whose gene was targeted is not known relative to the stage in the cascade, nor is the timepoint at which recombination begins after accumulation and translocation of CreERT2. This concern does not call into question their finding that the consequences of Notch manipulation change with progress along the cascade. But they need to be a little less definitive re: the timing of the normal molecular events. The other concern has to do with the interpretation of cell type percentages to define the effect. It is possible that cell death may sculpt the outcome, which is not the same as diversion to a different cellular differentiation pathway. While the outcome is essentially the same, the interpretation of molecular mechanism might well be confounded by unrecognized cell death. Some recognition of these interpretive concerns is warranted.
From the standpoint of the manuscript structure, the authors defer description of several of the supplementary results figures to the discussion, which is not best practice. The authors' adaptation of terminology from the course of neurogenesis in the main olfactory epithelium is strained and confusing.

© 2022. Published by The Company of Biologists under the terms of the Creative Commons Attribution License (https://creativecommons.org/licenses/by/4.0/).

“Progenitor” should be limited to dividing cells, of course. “Immediate neuronal precursor” should probably also designate dividing cells, if trying to use the term analogously to the main OE, and not post-mitotic daughter cells. The authors have generated a lovely molecular characterization along the way to full neuronal differentiation, why not use “early”, “mid” and “late” stages in neuronal differentiation on the way to full maturity to designate what is observed after Ki-67 disappears? That has the virtue of being clearer with respect to cell cycle behavior and the lifespan events. Also, from the scRNAseq data there is overlap between Neurogenin1/NeuroD1 expression and Ki67, indicating that not all of these are post-mitotic.
A couple of minor points should be noted: line 351 and following – NICD transduction/overexpression in the lesioned OE (Herrick, Guo et al) was assigned to GBCs not HBCs on the basis of the status of the epithelium at the time of vector infusion. Lines 308-309 – Bcl11b is duplicated in the sentence. Figure legend to SF1 and SF3 should note that the x-axis of the UMAP is compressed relative to Figure 1A (to minimize readers’ confusion). The different cell types identified in the scRNAseq UMAP analysis need to be labeled, not just the neurogenic cascade.

First revision

<u>Author response to reviewers' comments</u>

Reviewer 1 Advance summary and potential significance to field
In their manuscript, Katreddi et al. address the role of Notch signaling in the generation of vomeronasal sensory neurons. They performed scRNA sequencing of the vomeronasal sensory epithelium and performed a detailed computation analysis of the approx. 10,000 cells in the data set. They focused on the neural progenitor and immature neuronal stages to address fate segregation between apical and basal vomeronasal sensory neurons. Their analysis proposes that Notch signaling regulates the fate of a common Ascl1+ progenitor to V1R and V2R vomeronasal sensory neurons. They performed conditional Notch1 ablation from Ascl1+ cells and conditional gain of function of Notch1 signaling in Ascl1+ and Neurog1+ progenitors. Their results indicate that Notch1 can control the generation of V1R versus V2R (apical and basal) vomeronasal sensory neurons. The bioinformatics data is well presented and clear, although more description of the gene expression across all cells would be interesting as they focus on a very minor population here. The genetics data are interesting but need deepening and the mechanisms of action of Notch signaling in the control of vomeronasal sensory neuron fate are not formally show. The finding that Notch controls cell fate in what is claimed to be a committed progenitor is interesting.

Reviewer 1 Comments for the author
Main concerns
1.The bioinformatic analysis focusses on a small fraction of the cells in the UMAP analysis. It would be good to show the expression of the genes of interest on the entire cell populations shown in Figure 1A. These data can be included in the Supp Figures if needed but it will be interesting to see the distribution of mitotic cells, progenitor markers, neuronal and none neuronal cell population.

Feature plots of the genes related to neuronal progenitors, precursors, immature/mature neurons and sustentacular cells are now shown in whole Seurat object in supplementary figure1. We also added dot plot of genes used to identify different cell types in the scRNA-seq data. Besides this, an extensive analysis of differentially expressed genes between V1R+ apical and V2R+ basal neuronal population including additional scRNA seq cell libraries is offered in another article currently under consideration on a different journal. Including these in this manuscript would be redundant.

2. The Ascl1-CreER Notch1 LOF experiments should be expanded to look at earlier (1 dpi) and later time points. The analysis should also include cell death analyses and detailed analysis of proliferation.

We thank the reviewer for the suggestion. We now added Ascl1/Notch1fl/tdt KO animals at 1dpi, 3dpi and 14dpi stages. At 1 dpi and 3dpi, we did immunofluorescence against proliferation and cell death markers and compared to control tracing. At 14dpi, we repeated Meis2/Tfap2e similar to

© 2022. Published by The Company of Biologists under the terms of the Creative Commons Attribution License (https://creativecommons.org/licenses/by/4.0/).

7dpi and also included Gnai2 and Gnao1 analyses. See figure 5 & line 243 for 14dpi experiment and figure 7 & line 292 for 1 and 3dpi experiments.
3. The lineage tracing downstream of Ascl1-CreER is also superficial and should be extended.

We believe that in the current version of the manuscript, we provided all the information needed to understand and follow Ascl1 tracing. In fact, in figure 4, we followed the development of Ascl1+ cells in the window of 7 days showing that the majority of these cells leave the cell cycle within the analyzed time frame. This is an important information as we provide the first description of what is the time frame that it takes the transit amplifying progenitors in the VNO to become neurons. Moreover, as described in figure 5, we extended the analysis upto 7 and 14 days showing which proportion of the post mitotic cells acquire a specific cell type identity.

4. It would be good to show confirmatory data showing the distribution of Notch signaling components in the in the early vomeronasal epithelium - for example by in situ hybridization. The Notch1, Delta4 and NeuroD1 immunostaining imply that Notch signaling is restricted to the MZ and does not seem to be equal at all margins (see Figure 3).

We thank the reviewer for the interesting suggestion. However, the requested observations have been already published in Wakabayashi and Ichikawa 2007 and Benedito and Duarte 2005. We added these references in the text to guide the readers to find this specific analysis - see line 392

In synthesis during embryonic development, when proliferation occurs throughout the VNO, Notch and Dll4 are expressed throughout the epithelium. However, as neurons start to differentiate and proliferating niche start getting segregated to the marginal zone, Notch and delta appear to localize in the basal area of the marginal territories.

5. The Notch1 LOF should be extended to Neurog1-CreER deletion and analysis of cell fate.

We thank the reviewer for the suggestion. We now included Neurog1CreERT2 induced Notch1 LOF at 7dpi stage – see supplementary figure 5 and line 260.

6. The authors need to present some evidence that the loss of Notch1 on cell fate decisions is due to canonical Notch signaling. Currently, the evidence is rather week. Ideally the authors would perform similar loss of function experiments deleting Rbpj.

We thank the reviewer for the suggestion. We now included Ascl1CreERT2 induced Rbpj1 LOF experiment at 7dpi stage- see figure 6 and line 274.

7. The authors claim a reduced recombination of the Rosa-NICD locus to explain the reduced number of recombined cells. This may be true but needs to be controlled with a more extensive analysis of cell proliferation and cell death at early and later stages of development.

We agree with the reviewer that this would be an appropriate analysis to perform. However, it's not technically feasible, as the specific mouse line we use is known a) to recombine at low level (Cheung, Le Tissier et al. 2018); b) because GFP expression is driven by an IRES sequence, it results to be below detectability up to 1-2 days after recombination. Our attempts to respond to this request have failed.
In addition to this, reduced number of recombined GFP+ sustentacular cells in the Ascl1CreERT2/RNICD VNO could be due to direct differentiation of neuronal progenitors to sustentacular cells without any proliferation. From scRNA-seq analysis of multipotent horizontal basal cell lineage in the main olfactory epithelium, authors showed that stem cells directly differentiate without proliferation to sustentacular cells, whereas in order to transition to neurons, they first undergo proliferation followed by differentiation (Fletcher, Das et al. 2017).

8. The scRNA-seq is a snapshot of gene expression. This is particularly true for the expression of the Notch signaling and proneural genes which are presumably high dynamic and presumably also oscillatory. The predictions of lineage from the bioinformatics are purely predictions without experimental substantiation.
The authors should discuss this in their manuscript.

© 2022. Published by The Company of Biologists under the terms of the Creative Commons Attribution License (https://creativecommons.org/licenses/by/4.0/).

We accept to the reviewer1 comments. However, differently from bulk RNA seq, scrNA seq is less of a snapshot, but more like a sequence of frames that we can align to reconstruct dynamic expression of genes overtime. To stress this point, we included in the former fig 1, now in supplementary figure 1 a pseudotime reconstruction that shows the dynamics of gene expression from the stem cells to differentiated VSNs. Our analysis of gene expression along the developmental trajectories shows expression of Notch and delta at specific stages of the development of the VSNs. Moreover, our analysis points to differential activity of the Notch signaling pathway at different stages of VSNs differentiation and maturation.
Nonetheless, as suggested by the reviewer, we now added few lines in the text to better explain that our observations are focused on short developmental window of the VSNs. (See line 124)

9. The author claim a shift in apical to basal vomeronasal sensory neuronal fate due to manipulation of Notch signaling but this seems to be based on the expression of very few markers. The authors should substantiate a change in fate rather than a miss-expression of a few markers.

We thank the reviewer for pushing us to dig deeper. In the new version of the manuscript, we added 2 observations that we believe strengthen our conclusions in a considerable way. First, we analyzed if interfering with the Notch signaling could immediately affect Bcl11b expression that we know to be a downstream Notch target and a key regulator of basal differentiation for the VSNs (Li, Burke et al. 2010, Enomoto, Ohmoto et al. 2011). This experiment showed that as soon as 3 days post Notch ablation, Bcl11b expression in the proliferative cells significantly reduced- see figure 7 and line 302.

To further confirm that these cells were directed towards apical differentiation, we added the analysis for Gnao1 expression. As we predicted, loss of Notch resulted in a traced cells losing the basal specific G protein - see figure 5 and line 243.

Minor comment.
The authors discuss adult neurogenesis in the introduction but their analysis is purely developmental. This text should be adjusted not to cause confusion.

We only recently obtained scRNA seq data from perinatal animals (P10 age). We decided to add P10 data as a supplementary observation (see supplementary figure 4). These data confirm no differences in the mechanisms controlling neurogenesis across the ages. For this reason, we didn’t find a need to change the previously shown data of Figure1 and 2 obtained from older animals. We explained this in the text - see line 185.

A better description of the transgenic animals is needed. I suggest to include a scheme of the different alleles in the Supp Figures. This will help the general reader to understand the experimental approach.

We thank the reviewer for the suggestions. We indicated the mutated alleles in the experimental paradigm cartoons in each figure. We believe that these provide enough information to the reader to understand the experiments.

Are sustentacular cells a normal progeny of Ascl1+ progenitors? When are these cells normally generated in the vomeronasal sensory epithelium? This should be included in the introduction and expanded in the discussion.

How the sustentacular cells in the VNO forms is still a fuzzy area of exploration. We know from control Ascl1CreeRT2 tracing experiments that less than 1% Ascl1 progenitors give rise to Sust cells. Ascl1 seems, as expected to be limited to neurogenic cells. The Sus cells may likely come from cells with a higher level of stemness, we are currently investigating the origin and developmental trajectories of the Sus in the VNO, this will be part of follow-up paper.

In Figure 2 UMAP plots the authors use color-coded titles for the plots. This would be beneficial in Supp Figures 2, 4 and 5.

We thank the reviewer for the suggestion. We now added color-coded titles for the blend plots. See supplementary figure 3 and 6. We removed Ooep data and figure as suggested.

© 2022. Published by The Company of Biologists under the terms of the Creative Commons Attribution License (https://creativecommons.org/licenses/by/4.0/).

The authors need to state the statistical analysis used, SEM or SD and the n-value for all quantifications in the figures. For the numbers shown in the text, the authors need to add Sem or SDs and, when comparing control and mutants state the P-value and test used. In the methods section the authors claimed to have used t-Test for the statistics. Many of the analyses are percentages and t-Test is not the correct analysis unless the data have gone through an Arcsine transformation.

We thank the reviewer for the suggestion. All the numbers in the text are added with mean and sem values and mentioned in the text. All the percent values have been transformed to normal distribution using arcsine. Number of biological replicates added to figure legends. P values are shown in the plots. We indicated all statistical information in the figure legends and materials and methods section.

This reviewer does not think that the Ooep data really bring much to this manuscript as there is no experimental relevance presented for the Ooep expression.

We removed Ooep supplementary figure and also removed Ooep from discussion.

Reviewer 2 Advance summary and potential significance to field
This study from Forni and colleagues investigates the mechanisms used to specify apical vs. basal neuronal cell fates in the mouse vomeronasal organ (VNO) using a combination of single cell RNA-sequencing, lineage tracing, and genetic gain- and loss-of-function experiments. Overall this is a lovely study that demonstrates a role of notch-delta signaling in the specification of VNO neuronal types. While interesting in its own right, this is also a nice demonstration of how notch-delta signaling can impact such decisions once the neuronal fate has already been chosen, and not just in neuronal vs. non-neuronal cell fate decisions. I have a few concerns, none that are deal-breakers, that I would encourage the authors to consider before publication.

Reviewer 2 Comments for the author
1. On balance the single cell RNA-sequencing analysis looks solid. However by the number of cells in the lineage that are actually included in the main analysis is on the sparse side. I don't think that the authors' conclusions based on these experiments are very far off, given the results of subsequent Ascl1-cre lineage tracing and gain- and loss-of function experiments. However they might temper their conclusions as an analysis based on deeper sequencing might reveal some nuances.
We believe that though limited in number, our samples provided with the information that we confirmed histologically by immunolabelling and functionally with mouse genetic experiments. The use of scRNA sequencing in this work was directed to find potential mechanisms of action rather than for gene discoveries for which we agree a much deeper sequencing would be appropriate. However, we now included additional observations at P10 that confirmed as independent experiment that the expression we found at P60 is valid and conserved- see supplementary figure4.

2. Similarly, the lineage tracing results with NICD gain-of-function experiments are are a bit marginal to support the conclusions, considering the low efficiency of cre recombination/activation of the NICD allele.

NICD GOF results at Neurog1 stage have sufficient number of cells (even though less compared to Neurog1 control tracing) to compare to control data and come to conclusion.
On the other hand, for Ascl1CreERT2/RNICD condition, even though the recombined cells appear to be around a tenth of the R26tdTom control (that has a very high recombination), we believe that the number of cells we obtained is enough to support the shift from neuronal progenitors towards sustentacular cells. We removed figure 8F' plot as the analysis is based on less number of recombined cells.

3. The statistical analysis of lineage tracing and genetic analyses is not clear, or at least not easily accessible. How many mice were used in each figure panel and whether any sex differences were observed. Is an individual mouse consider a "sample" or "n?" Data in the figures are represented as percentages – absolute cell counts would be useful to provide as well. It also appears that the same

© 2022. Published by The Company of Biologists under the terms of the Creative Commons Attribution License (https://creativecommons.org/licenses/by/4.0/).

control data were used for multiple comparisons/figures; if so, this should be stated explicitly (apologies if I missed this).

We used 3 animals at every stage- considering each animal as one sample. Both males and females are included for GOF and LOF studies and we didn't find any difference in the phenotype. All statistical details are mentioned in the figure legends and materials and methods.
We now added traced neuronal numbers in the figure legends. We removed one bar plot where we used same control data (see comment#2 for reviewer2).

4. More information is needed about the cell clusters shown in the UMAP in Figure 1A – what cell types do the other clusters represent, and based on expression of what marker genes?

We now changed figure1A. Each color represents a cell type identified based on known gene expression. The markers used to identify the cell types are mentioned in the text (see line 104) and their gene expression shown as dot plot in supplementary figure 1.

Minor comments:

Page 2: typo (subject verb agreement, lines 46-47)
We changed the sentence- see line 44.

Page 4: How many mice were used? Age range? How many of each sex? The methods indicate P60 male mice. Please provide total number of mice and include that information here (in the main text) as well. Perhaps also add a note whether sex differences would or would not be expected.

Total 5 male mice at P60 age were included for scRNA-seq study. We included now this information in the results section- see line 96.

Page 6 (referring to the Bcl11b results shown in figure 2D vs. 2E-H): The Delta-Notch figures showing inverse expression are more convincing than the Bcl11b result alone, but they are convincing when considered as a whole. My question is whether the Bcl11b result could be supported with other data. There are nice antibodies for Bcl11b IHC that could be used in the GOF and LOF studies, for instance.

We thank the reviewer for the suggestion. We took the reviewer suggestions and purchased Bcl11b antibody. In the new version of the manuscript, we now included analysis of Bcl11b expression across proliferative precursors in control and Notch manipulated animals at 3dpi stage. Our Bcl11b data revealed a shift in the developmental trajectory even before AP-2ε+ expression- see line 302 and figure 7.

Page 11: Typo (line 309)
Corrected the typo- see line 396

Page 24 (referring to Figure 1A): A key or color scheme would help in the interpretation of this panel. Do the colors correspond to subclusters within each larger physical cluster? What do the other larger clusters correspond to? (see major concern #2 above)

We now changed figure1A. Each color represents to distinct identified cell types and the markers used to identify the cell types are mentioned in the text- see line 102.

Page 24 (referring to Figure 1B''): A color key is needed for this heatmap as well.

We now included color key- see figure 1C

Page 26 (referring to Figure 3): Perhaps include a directional in the first figure using IHC images (Fig. 3). Do these show a D-V axis or A-P axis?

We now added the axis in fig. 3

© 2022. Published by The Company of Biologists under the terms of the Creative Commons Attribution License (https://creativecommons.org/licenses/by/4.0/).

Page 27 (referring to figure 4): Does the central-peripheral gradient of tdTom expression correspond to a central-peripheral gradient of differentiation/maturation?

We believe central-peripheral gradient of tdTom expression corresponds to the extent of neurogenesis at the central vs marginal zones. It is known that from embryonic to postnatal and adult stages, neurogenesis varies at central vs marginal zones in the VNO. In the adult stages, neurogenesis is still reported to exist at the marginal zones of the sensory epithelium. We have Brdu unpublished data showing that newly formed VSNs remain in the marginal zones for more than 2 weeks after birth.

Page 28 and onward (referring to bar graphs): Could the authors include the corresponding raw numbers above each bar in 5D and D", ditto for other bar graphs (Figure 6F and F', Figure S4D)? See also major comment, above.

We now included raw numbers of neurons as suggested in the figure legends.

Page 28 (referring to Figure 5B,C): There seems to be a central-peripheral gradient of the effect of Notch on apical vs. basal fate. If this is the case, could the authors comment on it? Are there any known interactions with mitogens/signaling pathways with central-peripheral gradients?

From the scRNA seq data, we observed that Notch can be reexpressed in fully matured neurons. We believe that Notch mediated recombination in the central areas of the VNO might reflect re-expression of Notch in post-mitotic cells rather than labelling occurred during neurogenesis

Page 35 (referring to Figure S4): Add scale bars for S4 B-C''
We added scale bar now- see supplementary figure 6

Reviewer 3 Advance summary and potential significance to field
The manuscript by Katreddi et al investigates neuronal diversification in the Vomeronasal Organ (VNO) and describes the effect of manipulations of Notch signaling at stages within the neurogenic cascade. While the role of Notch1 in setting cell fate is certainly not new, the role of Notch in the VNO has not been explored and is important in understanding both VNO development and how Notch is functioning. The authors have very nicely characterized gene expression at discrete stages in the neurogenic cascade and related that to Notch.
The data will be extremely useful for designing future experiments to understand the contextualization of Notch signaling.

Reviewer 3 Comments for the author
This is a nice set of studies, but there are some issues with their approach and interpretation. The first potential problem, which the authors don't address, is the age disparity between the scRNAseq analysis in adult VNO and their localization and manipulation of Notch pathway components in the neonatal VNO. It is entirely possible that the two situation are not perfectly comparable and some justification needs to be offered.

Even though scRNA seq results were shown at adult stages (P60), we chose to validate and probe Notch signaling at early postnatal stages mainly due to more neurogenesis and the ability to get more traced neurons compared to adult stages. However, we took seriously the comment of the reviewer and therefore performed a new scRNA seq expression analysis at P10 age which is comparable to what used for genetic experiments in the paper. As we hypothesized, we didn't find any differences in the mechanisms controlling the establishment of the VSN dichotomy across ages.

We as previously explained to the response to reviewer1, we now included P10 feature plots in Supplementary figure 4. We decided not to change the main figures for our convenience. Access to P10 deposited data has been added to the manuscript.

Second, the analysis of the outcome of gene manipulation suffers from 2 potential flaws when trying to assign a role to Notch signaling at a specific point in the cascade. The authors are quite definitive in assigning the role to a specific cell type based on the driver for recombination. There are two potential problems with this interpretation. The rate of decay of the protein whose gene

© 2022. Published by The Company of Biologists under the terms of the Creative Commons Attribution License (https://creativecommons.org/licenses/by/4.0/).

was targeted is not known, relative to the stage in the cascade, nor is the timepoint at which recombination begins after accumulation and translocation of CreERT2. This concern does not call into question their finding that the consequences of Notch manipulation change with progress along the cascade. But they need to be a little less definitive re: the timing of the normal molecular events.

We agree with the reviewer that there are steps in the process of the recombination and decay of the protein prior recombination that we can't control. However, our new data show that Bcl11b which is a known target of Notch is already reduced in a significant fashion within proliferative cells 3 days after recombination- see figure 7 and line 302. As usual, there are aspects of the molecular manipulations in the individual cells that might be different in timing and efficiency. However, these are implicit flaws in invivo analysis not based on clones synchronized in a dish.

The other concern has to do with the interpretation of cell type percentages to define the effect. It is possible that cell death may sculpt the outcome, which is not the same as diversion to a different cellular differentiation pathway. While the outcome is essentially the same, the interpretation of molecular mechanism might well be confounded by unrecognized cell death. Some recognition of these interpretive concerns is warranted.

We thank the reviewer for rising this point. We went back to our mutants and analyzed the effects of Notch signaling loss of function on proliferation and cell death. By analyzing cells at 1dpi and 3 dpi stages that precede the 7dpi data shown earlier, we observed no changes in proliferation or cell death. However, we could see changes in the percentage of proliferative Bcl11b+ cells at 3dpi. These data support the idea of alternative programs rather than negative selection of cells - see figure 7.

From the standpoint of the manuscript structure, the authors defer description of several of the supplementary results figures to the discussion, which is not best practice.

As suggested by the reviewer1, we removed Ooep data from discussion. We also moved Foxn4 data to the results section in the new version – see line 260.

The authors' adaptation of terminology from the course of neurogenesis in the main olfactory epithelium is strained and confusing.
"Progenitor" should be limited to dividing cells, of course. "Immediate neuronal precursor" should probably also designate dividing cells, if trying to use the term analogously to the main OE, and not post-mitotic daughter cells. The authors have generated a lovely molecular characterization along the way to full neuronal differentiation, why not use "early", "mid" and "late" stages in neuronal differentiation on the way to full maturity to designate what is observed after Ki-67 disappears? That has the virtue of being clearer with respect to cell cycle behavior and the lifespan events. Also, from the scRNAseq data there is overlap between Neurogenin1/NeuroD1 expression and Ki67, indicating that not all of these are post-mitotic.

We thank the reviewer for the suggestions. From scRNA-seq analysis and lineage tracing data, we identified 2 populations of Neurod1+ cells. First one is Neurog1/Neurod1/Ki67 triple positive cells before the dichotomy, which we mentioned as immediate neuronal precursors similar to the nomenclature in the main olfactory epithelium. Second one is only Neurod1 positive and Neurog1/Ki67 double negative population expressed after the VSN dichotomy in both apical and basal branches. As this population is clearly segregated into different branches and don't express Ki67, In the new version, we are calling these Neurod1+ cells in apical and basal branches as post-mitotic apical and basal VSN precursors respectively- see figure 1, B, C and line 123.

A couple of minor points should be noted: line 351 and following – NICD transduction/overexpression in the lesioned OE (Herrick, Guo et al) was assigned to GBCs not HBCs on the basis of the status of the epithelium at the time of vector infusion.

We thank the reviewer for the suggestion. In Herrick, Guo et al 2018, two different experiments were shown. Firstly, as the reviewer mentioned, authors did viral infusion in rat model and suggested that NICD over expression at GBCs stage led them to differentiate towards sustentacular cells. Moreover, authors also performed conditional induction of NICD specifically in HBCs using

© 2022. Published by The Company of Biologists under the terms of the Creative Commons Attribution License (https://creativecommons.org/licenses/by/4.0/).

mouse genetics and showed that over expression of NICD specifically in HBCs also drive them to differentiate towards sustentacular cells during regeneration.

We now rephrased the sentence and mentioned both HBCs and GBCs - see line 454

Lines 308-309 – Bcl11b is duplicated in the sentence.
We corrected the sentence as suggested- see line 396

Figure legend to SF1 and SF3 should note that the x-axis of the UMAP is compressed relative to Figure 1A (to minimize readers' confusion).

We thank the reviewer for the suggestion. We corrected the UMAP plots- see supplementary figure 1 and supplementary figure 7.

The different cell types identified in the scRNAseq UMAP analysis need to be labeled, not just the neurogenic cascade.

We changed figure 1A now. We identified most of the clusters and also showed dot plot with the markers used to identify the cell types- see line 105, figure 1A and supplementary figure 1.

Reference:

Cheung, L., P. Le Tissier, S. G. Goldsmith, M. Treier, R. Lovell-Badge and K. Rizzoti (2018). "NOTCH activity differentially affects alternative cell fate acquisition and maintenance." Elife 7.

Enomoto, T., M. Ohmoto, T. Iwata, A. Uno, M. Saitou, T. Yamaguchi, R. Kominami, I. Matsumoto and J. Hirota (2011). "Bcl11b/Ctip2 controls the differentiation of vomeronasal sensory neurons in mice." J Neurosci 31(28): 10159-10173.

Fletcher, R. B., D. Das, L. Gadye, K. N. Street, A. Baudhuin, A. Wagner, M. B. Cole, Q. Flores, Y. G. Choi, N. Yosef, E. Purdom, S. Dudoit, D. Risso and J. Ngai (2017). "Deconstructing Olfactory Stem Cell Trajectories at Single-Cell Resolution." Cell Stem Cell 20(6): 817-830 e818.
Li, P., S. Burke, J. Wang, X. Chen, M. Ortiz, S. C. Lee, D. Lu, L. Campos, D. Goulding, B. L. Ng, G. Dougan, B. Huntly, B. Gottgens, N. A. Jenkins, N. G. Copeland, F. Colucci and P. Liu (2010). "Reprogramming of T cells to natural killer-like cells upon Bcl11b deletion." Science 329(5987): 85-89.

Second decision letter

MS ID#: DEVELOP/2021/200448

MS TITLE: Notch signaling determines cell-fate specification of the two main types of vomeronasal neurons of rodents.

AUTHORS: Raghu Ram Katreddi, Ed Zandro M Taroc, Sawyer M Hicks, Jennifer M Lin, Shuting Liu, Mengqing Xiang, and Paolo E Forni

I have now received the reports of two of the referees who reviewed the earlier version of your manuscript and I have reached a decision. The referees' comments are appended below, or you can access them online: please go to BenchPressand click on the 'Manuscripts with Decisions' queue in the Author Area.

The overall evaluation ismostlypositive and we would like to publish a revised manuscript in Development, provided that you satisfactorily address the remaining suggestions and comments of the referees.

© 2022. Published by The Company of Biologists under the terms of the Creative Commons Attribution License (https://creativecommons.org/licenses/by/4.0/).

Referee 1 still has a number of requests, some of which cannot be addressed within the time frame of a revision. Regarding the dataset used to produce Figure 1A, I agree with this referee that this data should be deposited in a repository before publication of the manuscript. Regarding the extension of the Ascl1-CreERT, Neurog1-CrER and RBPJk cKO analyses to P60, this does not seem feasible during the revision and can be omitted. Regarding the request to analyse cell proliferation and cell death in Rosa-NICD mice, you should clarify in the text that it was not possible to exclude changes in proliferation and cell death in these mice.

Please attend to all of the reviewers' comments in your revised manuscript and detail them in your point-by-point response. If you do not agree with any of their criticisms or suggestions explain clearly why this is so. If it would be helpful, you are welcome to contact us to discuss your revision in greater detail.

Reviewer 1

Advance summary and potential significance to field

In their manuscript, Katreddi et al. address the role of Notch signaling in the generation of vomeronasal sensory neurons. They performed scRNA sequencing of the vomeronasal sensory epithelium and performed a detailed computation analysis of the approx. 10,000 cells in the data set. Their analysis proposes that Notch signaling regulates the fate of a common Ascl1+ progenitor to V1R and V2R vomeronasal sensory neurons. Their results indicate that Notch1 can control the generation of V1R versus V2R (apical and basal)
vomeronasal sensory neurons. The finding that Notch controls cell fate in what is claimed to be a committed progenitor is interesting and would be useful for the community.

Comments for the author

In their revised manuscript, Katreddi et al. addressed some of my concerns and added new data. This has improved the manuscript somewhat. However, they deferred many of my concerns to another manuscript submitted elsewhere or reference to previous publications which do not address the main issues here.
This means the manuscript cannot be judged as a stand-alone report.
This reviewer believes that if data on DEGs are present in a manuscript, these genes need to be reported in the manuscript. This is not only so that the reader understands which genes are regulated and used in the analyses, but also so they can use this gene set to perform further analyses. I understand that the authors may have another manuscript in preparation (not cited) but the data should be made publicly-available for both manuscripts at the point of publication. If the authors do not want to do this here, they can deposit them in an accessible repository and refer to them there.
I still feel that, as the authors refer to adult neurogenesis and the scRNSseq analysis was performed on day 60 mice, the Ascl1-CreER analysis should have been extended to this endpoint at least in order to compare like with like.
Similarly, the Notch1 LOF by Neurog1-CreER-induced and the RBPj cKO deletion is welcomed but ideally the analyses should have been extended to the same time points analyzed in the Ascl1-CreER and through to postnatal day 60 at least.
This reviewer still feels that the authors could be missing important mechanisms by not addressing the NICD experiments fully. In my opinion, it is not a valid argument to say the model we used is bad and therefore we cannot analyze the experiment correctly – sorry then please use another model. I feel that cell proliferation and cell death after Notch1 forced activation is an important issue that should be addressed in this context.
With all respect, all RNAseq analyses are snapshots of gene expression at the time the RNA was isolated and it is extremely difficult and dangerous to predict prospective cell fates on the basis of data from a single time point without experimental lineage tracing. Dynamic gene expression means it is impossible to predict the fate a cell will adopt in the future, especially when the time frame of the determination and differentiation are not known.
I still feel it is important to show that sustentacular cells a normal progeny of Ascl1+ progenitors by performing simple Ascl1-CreERlineage tracing from P1 until at least P60? The question is whether all Sus cells are Ascl1-progeny.

© 2022. Published by The Company of Biologists under the terms of the Creative Commons Attribution License (https://creativecommons.org/licenses/by/4.0/).

Reviewer 2

Advance summary and potential significance to field

My concerns have been addressed by the authors.

Comments for the author

There may be a typo in line 188: is it meant to read “early postnatal age P10” rather than “P1”? If not, the specificity of P1 vs. P10 is confusing where “early postnatal” would be sufficient.

Second revision

Author response to reviewers' comments

Reviewer 1 Advance summary and potential significance to field
In their manuscript, Katreddi et al. address the role of Notch signaling in the generation of vomeronasal sensory neurons. They performed scRNA sequencing of the vomeronasal sensory epithelium and performed a detailed computation analysis of the approx. 10,000 cells in the data set. Their analysis proposes that Notch signaling regulates the fate of a common Ascl1+ progenitor to V1R and V2R vomeronasal sensory neurons. Their results indicate that Notch1 can control the generation of V1R versus V2R (apical and basal) vomeronasal sensory neurons. The finding that Notch controls cell fate in what is claimed to be a committed progenitor is interesting and would be useful for the community.

Reviewer 1 Comments for the author
In their revised manuscript, Katreddi et al. addressed some of my concerns and added new data. This has improved the manuscript somewhat. However, they deferred many of my concerns to another manuscript submitted elsewhere or reference to previous publications which do not address the main issues here.
This means the manuscript cannot be judged as a stand-alone report.

1. This reviewer believes that if data on DEGs are present in a manuscript, these genes need to be reported in the manuscript. This is not only so that the reader understands which genes are regulated and used in the analyses, but also so they can use this gene set to perform further analyses. I understand that the authors may have another manuscript in preparation (not cited) but the data should be made publicly-available for both manuscripts at the point of publication. If the authors do not want to do this here, they can deposit them in an accessible repository and refer to them there.

Our initial bioinformatic analyses in Figure1 and Supp figure1 (where we show multiple genes expression after the previous request of the reviewer) identified various cell types in the VNO and showed gene expression patterns from stem cell progenitors to immature VSNs. Notably nowhere in the paper we mention expression of additional genes that we do not show data of or we do not give access to. We therefore do not understand what the reviewer is referring to in their new request. Notably in the paper, when we focused on the differential gene expression between Bcl11b positive and negative cells we listed all the related genes of interest in Fig.2.
We did not mention anywhere about other differentially expressed genes between apical vs basal VSNs. However, while replying to reviewer1 main concerns #1 in our previous rebuttal, we mentioned that we included differentially expressed genes between apical vs basal VSNs in another article, however, these are not relevant for this story.
However, we now added the reference for the mentioned article on BiorxV, currently in revision at Elife, where more extensive DEGs between apical and basal VSNs are described and discussed – see line 187. In addition to this we granted public access to all the deposited raw data in GEO: GSM5719956; GSM5719957; GSE190330; GSM5764433

© 2022. Published by The Company of Biologists under the terms of the Creative Commons Attribution License (https://creativecommons.org/licenses/by/4.0/).

2. I still feel that, as the authors refer to adult neurogenesis and the scRNSseq analysis was performed on day 60 mice, the Ascl1-CreER analysis should have been extended to this endpoint at least in order to compare like with like.

In the last revision we included feature plots of Notch, Dll4 and other known genes like Meis2, Tfap2e in young (P10) and older (P60) mice. In the current version, we made a more extensive integrated scRNA seq analysis showing that the developmental trajectories of the VSNs is identical at p10 and p60. To make this very obvious we also included a merged feature plot in which we show the nearly perfect overlap between developing VSNs at different ages. We believe that as we will further discuss the addition of this data should address all the concerns of the reviewer.
Notably, we also show that 2 weeks after Tam injection nearly all (~99%) of the Ascl1 cells have differentiated (Line 202). To make this more clear, we now added to figure 4 an additional panel showing that 2 weeks after injections there are ~ 0.6% of proliferative cells. In the text we already explained the rationale behind our analysis (*Line 209). So, we do not really see the point in waiting for 60 days after injection when we know that between 1 and 2 weeks, 99% of the cells that were Ascl1 are now vomeronasal neurons.
Notably after the previous request of the reviewer we already included Ascl1CreERT2 induced Notch1 knockout observations at 7 and at 14 days post injection. Observations at the two different time points did not change the conclusion we obtained after observations at 7 days only.
Moreover, to compare P60 scRNA seq data with the genetic manipulations experiments, as the reviewer suggests, we would have to inject tamoxifen at P60 and collect mice approximately 4 weeks post injection; not collecting mice at P60 after injection at P1. Injecting at P60, waiting and analyze the data would take more than 3 months. As the VNO proliferation declines in the first month, this experiment would give a much smaller number of traced cells compared to what obtained from a perinatal mouse.

3. Similarly, the Notch1 LOF by Neurog1-CreER-induced and the RBPj cKO deletion is welcomed but ideally the analyses should have been extended to the same time points analyzed in the Ascl1-CreER and through to postnatal day 60 at least.
For the same reasons explained above, we do not really see the point in waiting for 60 days when the cells take around 2 weeks to differentiate. We believe that the data presented with all the different models are consistent and support our conclusions.

4. This reviewer still feels that the authors could be missing important mechanisms by not addressing the NICD experiments fully. In my opinion, it is not a valid argument to say the model we used is bad and therefore we cannot analyze the experiment correctly – sorry then please use another model. I feel that cell proliferation and cell death after Notch1 forced activation is an important issue that should be addressed in this context.

As suggested by the editor, we now better explained in the text why in Rosa-NICD mice it was not possible to evaluate changes in proliferation and cell death at early stages. We also mentioned that we cannot exclude a role for proliferation and cell death in the observed phenotype—see line 439.

5. With all respect, all RNAseq analyses are snapshots of gene expression at the time the RNA was isolated and it is extremely difficult and dangerous to predict prospective cell fates on the basis of data from a single time point without experimental lineage tracing. Dynamic gene expression means it is impossible to predict the fate a cell will adopt in the future, especially when the time frame of the determination and differentiation are not known.

The VNO generates new neurons throughout life. Our single cell seq are thousands of “snapshots” representing individual non-synchronized cells at different stages of development from stem cells to differentiated neurons, not one snapshot. These “snapshots” can be ordered based on differential RNA expression to generate pseudo-temporal reconstruction. Based on the previous comment and the insistence of the reviewer on this point, we included a comparative Single cell seq data analysis of cells obtained from P10 and P60 animals. These data show that the VSNs have similar developmental trajectories regardless of the age. All sets of data are deposited in the GEO and available to the community, accession numbers are available in the text.
Respectfully, we still do not understand what the concern of the reviewer is here. We used the scRNA seq data to identify genes differentially express during the process of differentiation of the VSNs, event that our sc Cell data allowed to identify in its dynamics. Based on these data we

© 2022. Published by The Company of Biologists under the terms of the Creative Commons Attribution License (https://creativecommons.org/licenses/by/4.0/).

generated the hypothesis that the differentiation dichotomy of the VSNs is dictated by Notch signaling. We tested our hypothesis in vivo using multiple mouse models, we did follow the cell fate of the traced cells, and we performed conditional mutagenesis. As described above based on our data we know that the timeframe we analyzed (2 weeks after recombination) is sufficient to determine the final fate of the cells.

6. I still feel it is important to show that sustentacular cells a normal progeny of Ascl1+ progenitors by performing simple Ascl1-CreERlineage tracing from P1 until at least P60? The question is whether all Sus cells are Ascl1-progeny.

We already included Ascl1-CreERlineage tracing from P1 to P15. From the Ascl1CreERT2 lineage traced analysis at P1 to 7dpi, we showed that more than 99% of traced cells become VSNs and only less than 1% of the Ascl1 traced cells are Sustentacular cells. We believe that nothing really happens 2 weeks after recombination as all the cells are differentiated. Our data point to the fact that in control animals a very small number of sustentacular cells appears to be sportive for Ascl1 lineage.

Reviewer 2 Advance summary and potential significance to field
My concerns have been addressed by the authors.

Reviewer 2 Comments for the author
There may be a typo in line 188: is it meant to read “early postnatal age P10” rather than “P1”? If not, the specificity of P1 vs. P10 is confusing where “early postnatal” would be sufficient.

We agree that the wording was somehow confusing we removed P1 and left early postnatal – see line 184

Third decision letter

MS ID#: DEVELOP/2021/200448

MS TITLE: Notch signaling determines cell-fate specification of the two main types of vomeronasal neurons of rodents.

AUTHORS: Raghu Ram Katreddi, Ed Zandro M Taroc, Sawyer M Hicks, Jennifer M Lin, Shuting Liu, Mengqing Xiang, and Paolo E Forni
ARTICLE TYPE: Research Article

I am delighted to tell you that your manuscript has been accepted for publication in Development, pending our standard ethics checks.

© 2022. Published by The Company of Biologists under the terms of the Creative Commons Attribution License (https://creativecommons.org/licenses/by/4.0/).
